# Label‐Free Quantitative Proteomic Analysis of Differentially Expressed Membrane Proteins of Pulmonary Alveolar Macrophages Infected with Highly Pathogenic Porcine Reproductive and Respiratory Syndrome Virus and Its Attenuated Strain

**DOI:** 10.1002/pmic.201700101

**Published:** 2017-11-24

**Authors:** Zehui Qu, Fei Gao, Liwei Li, Yujiao Zhang, Yifeng Jiang, Lingxue Yu, Yanjun Zhou, Hao Zheng, Wu Tong, Guoxin Li, Guangzhi Tong

**Affiliations:** ^1^ Department of Swine Infectious Diseases Shanghai Veterinary Research Institute Chinese Academy of Agricultural Sciences Shanghai P. R. China; ^2^ Jiangsu Co‐innovation Center for Prevention and Control of Important Animal Infectious Diseases and Zoonoses Yangzhou P. R. China

**Keywords:** attenuated, highly pathogenic, infection, label‐free quantitative proteomics, membrane proteins

## Abstract

Significant differences exist between the highly pathogenic (HP) porcine reproductive and respiratory syndrome virus (PRRSV) and its attenuated pathogenic (AP) strain in the ability to infect host cells. The mechanisms by which different virulent strains invade host cells remain relatively unknown. In this study, pulmonary alveolar macrophages (PAMs) are infected with HP‐PRRSV (HuN4) and AP‐PRRSV (HuN4‐F112) for 24 h, then harvested and subjected to label‐free quantitative MS. A total of 2849 proteins are identified, including 95 that are differentially expressed. Among them, 26 proteins are located on the membrane. The most differentially expressed proteins are involved in response to stimulus, metabolic process, and immune system process, which mainly have the function of binding and catalytic activity. Cluster of differentiation CD163, vimentin (VIM), and nmII as well as detected proteins are assessed together by string analysis, which elucidated a potentially different infection mechanism. According to the function annotations, PRRSV with different virulence may mainly differ in immunology, inflammation, immune evasion as well as cell apoptosis. This is the first attempt to explore the differential characteristics between HP‐PRRSV and its attenuated PRRSV infected PAMs focusing on membrane proteins which will be of great help to further understand the different infective mechanisms of HP‐PRRSV and AP‐PRRSV.

## 1 Introduction

Porcine reproductive and respiratory syndrome virus (PRRSV) has been a leading economically significant viral pathogen of swine worldwide for almost 28 years.^1^, ^2^, ^3^ PRRSV, equine arteritis virus, simian hemorrhagic fever virus, and lactate dehydrogenase‐elevating virus are members of the family *Arteriviridae*.^4^, ^5^ PRRSV is a positive‐sense, single‐stranded, RNA virus with a full‐length genome of 15 kb that has a 5′ cap and a 3′ poly (A) tail.^4^, ^6^, ^7^, ^8^ PRRSV was first reported in the United States in the late 1980s.^9^ In 2006, several large‐scale, severe outbreaks of highly pathogenic (HP) atypical PRRSV (HP‐PRRSV) were reported in China and neighboring Asian countries.^2^, ^3^, ^10^ Reported HP‐PRRSV morbidity and mortality rates were much higher than previous pandemic PRRSV strains and associated with more severe clinical presentations and higher rectal temperature (>41 °C).^11^ The emergence of HP‐PRRSV has caused great economic loss to the swine industry in China and made preventing and controlling PRRSV outbreaks difficult. Therefore, elucidating the causes of the greater virulence of PRRSV and the differences between the HP and attenuated pathogenic (AP) strains has become even more important. To this end, several trials have been conducted to identify virulence factors; these studies have resulted in some successes.^12^, ^13^ However, changes in virulence and pathogenic mechanisms are difficult to discern. Other than virulence in vivo, many distinctions in the biological aspects of HP‐PRRSV and AP‐PRRSV have been noted, such as in viral binding and entry into pulmonary alveolar macrophages (PAMs).

**Significance of the study**

Membrane proteins (MPs) of PAMs infected by highly proteomic (HP)‐ and attenuated proteomic (AP)‐PRRSV have been elucidated by LC‐MS/MS for label‐free quantitative proteomics. Ninety‐five differentially expressed proteins were identified and characterized. The most significant difference in the biological process between PAMs infected with HP‐ and AP‐PRRSV is the metabolic process. Most different molecular functions were classified as binding and catalytic activities. Cellular component categories showed that 26 differentially expressed proteins were confirmed as MPs based on the annotation of UniProt database, such as RAP2A, VCL (Vinculin), IFITM3 function in cell–cell junctions, ERK signaling pathway, G protein signaling pathways, biotic stimulus, and so on. Among them, VCL is a kind of F‐actin‐ binding protein which is involved in cell‐matrix adhesion and cell–cell adhesion in humans. It was demonstrated that over expression of VCL could inhibit the replication of both HP‐PRRSV and the attenuated PRRSV in the mRNA level. There were obvious differences in the inhibiting ability for HP‐PRRSV and its attenuated strain. This is the first attempt to explore the differential characteristics between HP‐PRRSV and its attenuated PRRSV‐infected PAMs focusing on MPs which will be of great help to further understand the different infective mechanisms of HP‐PRRSV and AP‐PRRSV.

PRRSV exhibits highly restricted cell tropism both in vivo and in vitro.^14^ The virus can be detected only in well‐differentiated macrophages of lungs, lymph nodes, Peyer's patches, spleen, tonsils, and thymus. PAMs are the main target cells of PRRSV.^15^ PRRSV can also replicate in vitro in the African green monkey kidney cell line MA‐104 and its derivatives, MARC‐145 and CL‐2621, which are considered permissive cell lines for PRRSV.^16^, ^17^ Reportedly, PRRSV targets cellular membrane proteins (MPs) and enters target cells through receptor‐mediated endocytosis during viral infection.^18^ Studies have investigated possible mechanisms employed by PRRSV to infect PAMs.^19^

Elucidating the characteristics of viruses and interactions between viruses and host cells is increasingly important. However, exploring individual proteins within the many proteins in cells is difficult. Nonetheless, gradual advancements have come through use of proteomic techniques. Of these, MS‐based quantitative proteomic techniques offer the advantage of better accuracy and sensitivity, and have been widely used to analyze host cell responses to viral infection. Among them, liquid chromatography‐tandem MS (LC‐MS/MS) for label‐free quantitative proteomics (LFQP) is an important mass spectrometric tool to detect and quantify large amounts of proteins.^20^ Compared with quantitative proteomics using stable isotope labeling such as stable isotope labeling by amino acids in cell culture and isobaric tags for relative and absolute quantitation, LFQP detects greater amounts of proteins and important signaling pathways and networks. The aim of this study was to determine potentially different infection mechanisms used by the HP strain vHuN4^10^ and its derivative attenuated strain vHuN4‐F112^21^, ^22^ using LFQP.

## 2 Experimental Section

### 2.1 Ethics Statement

The animal study protocols were approved by the Animal Care and Use Committee of Shanghai Veterinary Research Institute, Chinese Academy of Agricultural Sciences.

### 2.2 Viruses and Cell Cultures

HP‐PRRSV strain vHuN4^3^, ^10^ at a titer of 10^5^ 50% tissue culture infective dose (TCID_50_) mL^−1^ and cell‐passaged attenuated virus strain vHuN4‐F112 (AP‐PRRSV)^21^, ^22^ at a titer of 10^5^ TCID_50_ mL^−1^ were stored as viral stocks. Porcine circovirus 2, classical swine fever virus, PRRSV antibody, and antigen‐free 15‐day‐old piglets were used. Animals were sacrificed in accordance with the ethics statement. Lungs were dissected and lavaged with PBS (PBS; Life Technologies, Inc., Gibco/BRL Division, Grand Island, NY, USA) supplemented with 1% penicillin–streptomycin (Gibco/BRL), then centrifuged at 1000 × *g* for 5 min, resuspended in PBS, centrifuged, and resuspended in PBS. PAMs were collected in Roswell Park Memorial Institute (RPMI) 1640 medium (Gibco/BRL) containing 10% fetal bovine serum (Gibco/BRL)^23^ and incubated in 10 cm dishes (Corning, Inc., Corning, NY, USA) for 12 h at 37 °C in a 5% CO_2_ atmosphere.

### 2.3 Virus Inoculation

After PAMs were washed with PBS three times, dead and nonadherent cells were removed when confluency exceeded 95%. Three dishes were inoculated with vHuN4 and another three with vHuN4‐F112 at multiplicity of infection 1. An additional three dishes were inoculated with DMEM (Gibco‐BRL) as a blank control. All dishes were incubated at 37 °C in an atmosphere of 5% CO_2_, as described previously.^13^ After incubation for 1 h, inocula were discarded and PAMs were washed with PBS three times. Cell monolayers in all dishes were overlaid with RPMI‐1640 medium containing 2% fetal bovine serum and incubated at 37 °C in a 5% CO_2_ atmosphere for 24 h.

### 2.4 Extraction of MPs of PAMs

PAMs were digested with 2 mL 0.25% trypsin–ethylenediaminetetraacetic acid solution (Gibco/BRL), collected by gently pipetting, centrifuged at 1000 × *g* for 5 min and lysed using the ProteoExtract Transmembrane Protein Extraction Kits (NOVAGEN, EMD Biosciences, Inc., Madison, WI, USA),^24^, ^25^ according to the manufacturer's instructions. Cells were resuspended in Extraction Buffer 1 and protease inhibitor cocktail, incubated for 10 min at 4 °C with gentle agitation, and centrifuged at 1000 × *g* for 5 min at 4 °C. After removing supernatants, pellets were resuspended in 0.2 mL Extraction Buffer 2A and protease inhibition cocktail, incubated for 45 min at room temperature with gentle agitation, and centrifuged at 16 000 × *g* for 15 min at 4 °C. Supernatants were precipitated with 1 mL acetone and centrifuged at 12 000 × *g* for 10 min. After evaporating to dryness, 150 μL SDT buffer (4% sodium dodecyl sulfate, 100 mm Tris/HCl at pH 7.6, 0.1 m dithiothreitol) was added and mixtures were heated in boiling water for 5 min. After centrifugation, supernatants were collected and quantified with a BCA Protein Assay Kit (Bio‐Rad, USA).

### 2.5 Label‐Free Quantitative ProteomicsLFQP was performed as shown in Fig. 1

#### 2.5.1 Protein Digestion

Digestion of protein (250 μg for each sample) was performed according to the FASP (Filter‐Aided Sample Preparation) procedure. Briefly, the detergent, DTT and other low‐molecular‐weight components were removed using 200 μL UA buffer (8 m Urea, 150 mm Tris‐HCl pH 8.0) by repeated ultrafiltration (Microcon units, 10 kD) facilitated by centrifugation. Then 100 μL 0.05 m iodoacetamide in UA buffer was added to block reduced cysteine residues and the samples were incubated for 20 min in darkness. The filter was washed with 100 μL UA buffer three times and then 100 μL 25 mm NH_4_HCO_3_ twice. Finally, the protein suspension was digested with 3 μg trypsin (Promega) in 40 μL 25 mm NH_4_HCO_3_ overnight at 37 °C, and the resulting peptides were collected as a filtrate. The peptide content was estimated by UV light spectral density at 280 nm using an extinctions coefficient of 1.1 of 0.1% (g L^−1^) solution that was calculated on the basis of the frequency of tryptophan and tyrosine in vertebrate proteins.^26^

**Figure 1:**
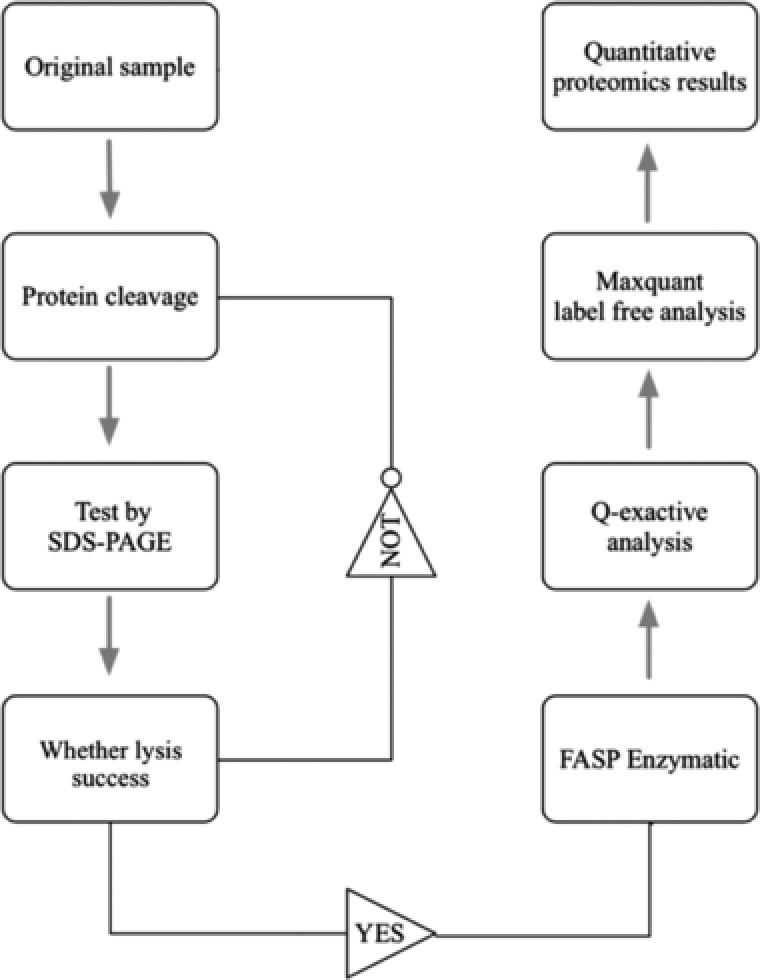
LFQP protocol used in this study.

#### 2.5.2 LC‐MS/MS Analysis

The peptide of each sample was desalted on C18 Cartridges (Empore SPE Cartridges C18 (standard density), bed id 7 mm, volume 3 mL, Sigma), then concentrated by vacuum centrifugation and reconstituted in 40 μL of 0.1% (v/v) trifluoroacetic acid. MS experiments were performed on a Q Exactive mass spectrometer that was coupled to Easy nLC (Proxeon Biosystems, now Thermo Fisher Scientific). Five microgram peptide was loaded onto a C18‐reversed phase column (Thermo Scientific Easy Column, 10 cm long, 75 μm inner diameter, 3 μm resin) in buffer A (2% acetonitrile and 0.1% Formic acid) and separated with a linear gradient of buffer B (80% acetonitrile and 0.1% Formic acid) at a flow rate of 250 nL min^−1^ controlled by IntelliFlow technology over 120 min. MS data was acquired using a data‐dependent top ten method dynamically choosing the most abundant precursor ions from the survey scan (300–1800* m/z*) for HCD fragmentation. Determination of the target value is based on predictive automatic gain control. Dynamic exclusion duration was 25 s. Survey scans were acquired at a resolution of 70 000 at *m/z* 200 and resolution for HCD spectra was set to 17 500 at *m/z* 200. Normalized collision energy was 30 eV and the underfill ratio, which specifies the minimum percentage of the target value likely to be reached at maximum fill time, was defined as 0.1%. The instrument was run with peptide recognition mode enabled. MS experiments were performed triply for each sample.^27^

#### 2.5.3 Sequence Database Searching and Data Analysis

The MS data were analyzed using MaxQuant software version 1.3.0.5. MS data were searched against the uniprot Sus scrofa sequence database (including 34 253 sequences downloaded on 12/27/2014). An initial search was set at a precursor mass window of 6 ppm. The search followed an enzymatic cleavage rule of Trypsin/P and allowed maximal two missed cleavage sites and a mass tolerance of 20 ppm for fragment ions. Carbamidomethylation of cysteines was defined as fixed modification, while protein N‐terminal acetylation and methionine oxidation were defined as variable modifications for database searching. The cutoff of global false discovery rate for peptide and protein identification was set to 0.01. Label‐free quantification was carried out in MaxQuant as previously described. Protein abundance was calculated on the basis of the normalized spectral protein intensity (LFQ intensity).

All statistical analyses were performed using unpaired *t*‐tests. A *p*‐value <0.05 and ratio >2 or <0.5 were considered to indicate significant differences. Gene Ontology (GO) annotation and functional classification of identified proteins was with Blast2GO ver. V2.6.2 with the current public database b2g_aug 12 (http://www.Blast2go.com). Identified proteins were classified using Blast2go steps under default parameters: blast, mapping, and annotation. Protein–protein interaction networks were analyzed using (String software string‐db.org/) . Confidence view was assigned a score of 0.4, indicating medium confidence.

### 2.6 Western Blots

Samples of PRRSV‐infected and DMEM‐inoculated PAMs were lysed at 24 h post infection and protein concentrations were determined. Samples (20 μg) were separated by 12% SDS‐PAGE and transferred to 0.22 μm nitrocellulose membranes (Bio‐Rad Laboratories, Hercules, CA, USA). Membranes were blocked with 5% skim milk in Tris‐buffered saline containing 0.05% Tween‐20 and incubated overnight at 4 °C with monoclonal antibodies against heat shock protein 70 (HSP70; ab5439; Abcam plc, Cambridge, UK) or KDEL receptor (ab69659; Abcam plc). After washing three times, membranes were incubated at 37 °C for 60 min with horseradish peroxidase‐conjugated anti‐mouse IgG or anti‐rabbit IgG (Abcam plc). Detection used chemiluminescence luminal reagents (Pierce Biotechnology, Waltham, MA, USA).

### 2.7 Expression Vector Construction and Transfection

The porcine vinculin (VCL) were amplified from the cDNA obtained from PAM cells. Restriction enzyme sites were incorporated into the primer sequences to facilitate molecular cloning. PCR products were cloned into the pCAGGS vector to produce the porcine VCL expression vector. For transfection, cells were seeded in 6‐well plates (Corning) and transfected at 70–80% confluency with respective constructed plasmids DNA by using Lipofectamine 3000 (Life Technologies), according to the manufacturer's instructions. The HP‐HuN4 or HuN4‐F112 was infected in MARC‐145 cells at 36 h post transfection. Empty vector transfection samples served as controls in the experiment.

### 2.8 RNA Extraction, cDNA Synthesis, and Real‐time RT‐PCR

At 48 h post infection, total RNAs of PRRSV‐infected cells were extracted using the RNeasy mini kit (Qiagen), the viral RNAs in supernatants were isolated using QIAamp Viral RNA Mini kit (Qiagen), according to the instruction manual. All the isolated RNAs were used as the template for synthesis of first‐strand cDNA by RT‐PCR using RT primed by oligo (dT)_18_ primer using the PrimeScript RT Master Mix (Perfect Real Time, TaKaRa), according to the manufacturer's instructions. Then the cDNA templates were quantified using PRRSV‐specific real‐time RT‐qPCR.^28^

## 3 Results

### 3.1 Label‐Free LC‐MS/MS for Quantitative Analysis of MPs of PAMs Infected with PRRSVs of Different Virulence

**Figure 2:**
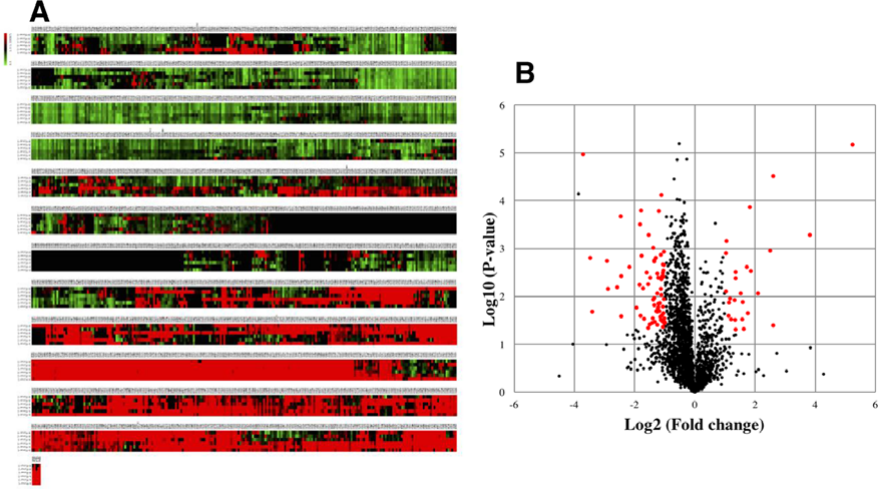
A) Heatmap of detected proteins of PAMs inoculated with AP‐PRRSV and HP‐PRRSV. Left annotations, samples after loading three times. Cube colors, levels of differentially expressed proteins: depth of red, degree of upregulation; depth of green, degree of downregulation; black, equal to the average amount detected in the blank control (DMEM). Protein names are represented by UniProt accession numbers. B) Volcano plot showing levels of differentially expressed proteins detected in HP‐PRRSV‐infection and AP‐PRRSV‐infection groups. X axis, mean log2 (ratio of fold change); y axis, log10 (*p*‐value); red balls, differentially expressed proteins between the two groups (*p* < 0.05 and ratio >2 or <0.5).

**Figure 3:**
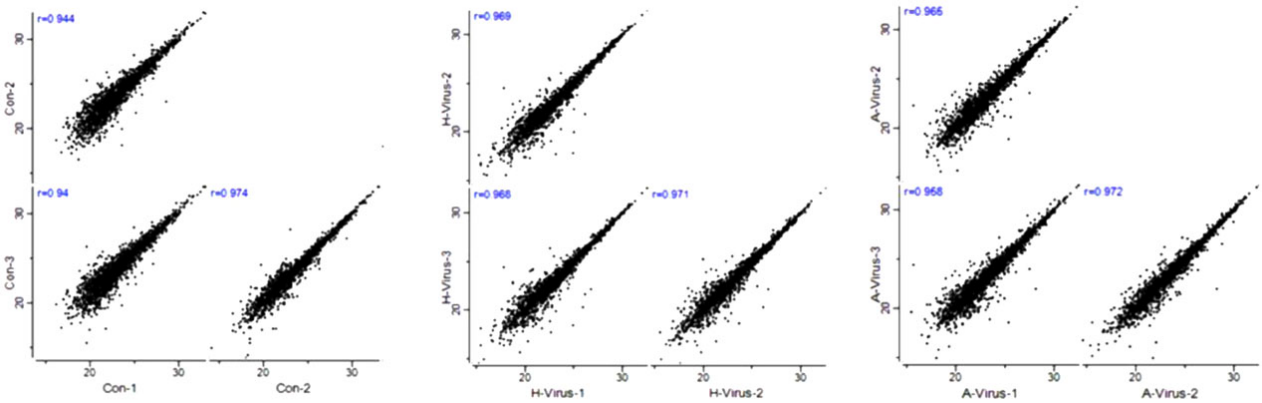
Correlation analysis. A) Three time‐loaded control; B) HP‐PRRSV samples; C) AP‐PRRSV samples. X and Y axes are log2 values (LFQ intensity).^87^

A total of 2849 proteins were detected by LFQP and are displayed in a heatmap (Figure 2). Statistical significance was determined using unpaired *t*‐tests. For all tests, a *p*‐value of <0.05 and ratio of >2 or <0.5 was considered to indicate a significant difference. Glyceraldehyde 3‐phosphate dehydrogenase was the internal normalization control and the ratio of glyceraldehyde 3‐phosphate dehydrogenase between HP and AP‐PRRSV was 0.95 ± 0.55. The “only one exists” group indicated that proteins were detected only in one group but not another group due to low expression level (Table 1). Correlation analysis indicated good repeatability of the technology (Figure 3).

**Table 1:** Overview of differentially expressed proteins

ISSUE	QUANTITATIVE DIFFERENCE	“ONLY ONE EXISTS”
HP‐PRRSV versus CONTROL	256	158
AP‐PRRSV versus CONTROL	195	90
HP‐PRRSV versus AP‐PRRSV	52	43

*p* <0.01 and ratio >2 or <0.5 indicated quantitative difference between two groups. The “only one exits” group indicated that proteins were detected three times in one, but not in the other group. HP‐PRRSV group means proteins identified in the HP‐PRRSV infected PAMs, while AP‐PRRSV was proteins detected in the attenuated pathogenic PRRSV infected PAMs. Control was displayed as the mock.

A total of 95 differentially expressed proteins were identified (Table 2). A control group was used to exclude false‐positive interference. Data from the control group was also used to elucidate functions of target proteins. Differentially expressed proteins between control and HP‐PRRSV‐infected cells (Con/HP) and the control and AP‐PRRSV‐infected cells (Con/AP) are in Supporting Information.

**Table 2:** Statistics analysis of the 95 differentially expressed proteins between HP‐PRRSV group and AP‐PRRSV

No.	Description	UniProt accession	#GOs	*t*‐TEST(AP/HP‐PRRSV)	HP/AP‐PRRSV	No.	Description	UniProt accession	#GOs	*t*‐TEST(AP/HP‐PRRSV)	HP/AP‐PRRSV
1	nuclear autoantigen sp‐100 isoform 4	tr|R4L6V8|R4L6V8_PIG	31	7.12 × 10^–05^	0.069	49	Ribonuclease kappa	tr|F1RF51|F1RF51_PIG	3	1.79E‐03	0.394
2	PREDICTED: collectin‐12	tr|K9J4M9|K9J4M9_PIG	8	2.43 × 10^−03^	3.294	50	af4 fmr2 family member 4 isoform x1	tr|F1RI16|F1RI16_PIG	0	2.41E‐03	0.222
3	Enhancer of mRNA‐decapping protein 3	tr|F1SIE7|F1SIE7_PIG	4	1.84 × 10^−03^	0.39	51	Kinesin light chain 4 isoform x3	tr|F1RRN1|F1RRN1_PIG	3	3.08E‐05	6.048
4	Low quality protein: atp‐binding cassette sub‐family b member mitochondrial	tr|F1SR85|F1SR85_PIG	16	2.09 × 10^−04^	0.182	52	Coatomer subunit beta‐like protein	tr|I3LAW4|I3LAW4_PIG	4	8.62E‐03	4.298
5	u1 small nuclear ribonucleoprotein 70 kda isoform x1	tr|I3LNE5|I3LNE5_PIG	9	7.61× 10^−05^	0.465	53	Heat shock factor‐binding protein 1	tr|I3LU44|I3LU44_PIG	1	N/A	vH+,vA–
6	Adenylosuccinate lyase	tr|D2KPI8|D2KPI8_PIG	13	3.15 × 10^−04^	0.283	54	m‐phase‐specific plk1‐interacting protein	tr|F1SSC4|F1SSC4_PIG	4	N/A	vH+,vA–
7	Acyl‐protein thioesterase 1	tr|F1RSG9|F1RSG9_PIG	7	7.06 × 10^−04^	2.062	55	Ras‐related protein rap‐2b	sp|Q06AU2|RAP2A_PIG	20	N/A	vH+,vA–
8	IFN regulatory factor 3	sp|Q764M6|IRF3_PIG	18	1.57 × 10^−03^	0.09	56	Histone ‐like	tr|F2Z5K9|F2Z5K9_PIG	16	N/A	vH+,vA–
9	ADP‐ribosylation factor gtpase‐activating protein 2 isoform x1	tr|F1SIB9|F1SIB9_PIG	4	4.03 × 10^−03^	0.357	57	Leucine‐rich repeat interacting protein‐1	tr|M1FV56|M1FV56_PIG	4	N/A	vH+,vA–
10	Isoform cra_b	tr|Q29194|Q29194_PIG	0	1.11 × 10^−03^	5.683	58	DNAJ homolog subfamily b member 2 isoform x1	tr|F1SR79|F1SR79_PIG	17	N/A	vH+,vA–
11	Hematological and neurological expressed 1‐like protein	tr|I7KJP5|I7KJP5_PIG	2	2.85 × 10^−03^	0.483	59	Heart fatty acid‐binding protein	sp|O02772|FABPH_PIG	7	N/A	vH+,vA–
12	Raftlin‐ partial	tr|I3LJL8|I3LJL8_PIG	16	4.13 × 10^−03^	2.554	60	E3 ubiquitin‐protein ligase trim56	tr|A0A077ETG0|A0A077ETG0_PIG	11	N/A	vH+,vA–
13	Nucleoporin seh1‐like isoform x2	tr|I3LR40|I3LR40_PIG	6	1.79 × 10^−03^	0.133	61	Programmed cell death protein 5	tr|F1RNX2|F1RNX2_PIG	6	N/A	vH+,vA–
14	Aconitate mitochondrial	sp|P16276|ACON_PIG	6	3.83 × 10^−03^	0.469	62	Heat shock transcription factor 1	tr|F1RSM7|F1RSM7_PIG	21	N/A	vH+,vA–
15	Thiosulfate sulfurtransferase	tr|F1SKL2|F1SKL2_PIG	8	6.82 × 10^−03^	0.305	63	*N*‐alpha‐acetyltransferase 50 isoform x1	tr|F2Z5Q7|F2Z5Q7_PIG	9	N/A	vH+,vA–
16	Lysc1_pig ame: full = lysozyme c‐1 ame: full = ‐beta‐n‐Acetylmuramidase c	sp|P12067|LYSC1_PIG	9	1.64 × 10^−04^	0.434	64	Golgin subfamily a member 2	tr|I3L9Y2|I3L9Y2_PIG	4	N/A	vH+,vA–
17	*n*‐acylethanolamine‐hydrolyzing acid amidase	tr|F1RYU7|F1RYU7_PIG	5	6.94 × 10^−03^	0.455	65	TBC1 domain family member 2a	tr|F1SSG2|F1SSG2_PIG	2	N/A	vH+,vA–
18	Dipeptidyl peptidase 2	tr|I3LU34|I3LU34_PIG	0	3.18 × 10^−03^	0.33	66	IFN‐induced protein with tetratricopeptide repeats 2	tr|J7FJH8|J7FJH8_PIG	6	N/A	vH+,vA–
19	Paraspeckle component 1	tr|F1RN28|F1RN28_PIG	4	2.15 × 10^−03^	0.476	67	Uridine 5 ‐monophosphate synthase	tr|I3LVD6|I3LVD6_PIG	0	N/A	vH+,vA–
20	ADP ATP translocase 3	sp|Q6QRN9|ADT3_PIG	16	6.47 × 10^−03^	0.167	68	Thymocyte nuclear protein 1‐like	tr|F1S6C1|F1S6C1_PIG	0	N/A	vH+,vA–
21	Peptidyl‐prolyl cis‐trans isomerase b	tr|F1S0A2|F1S0A2_PIG	13	1.56 × 10^−03^	0.406	69	Ataxin‐2 isoform x2	tr|F1RMZ0|F1RMZ0_PIG	17	N/A	vH+,vA–
22	Translation machinery‐associated protein 7	tr|F2Z5V1|F2Z5V1_PIG	3	1.63 × 10^−04^	0.29	70	AP‐1 complex subunit beta‐1 isoform x3	tr|F1RFI2|F1RFI2_PIG	4	N/A	vH+,vA–
23	Nucleolar and coiled‐body phosphoprotein 1 isoform x1	tr|F1S8T1|F1S8T1_PIG	4	4.06 × 10^−03^	0.429	71	Vinculin isoform x1	sp|P26234|VINC_PIG	26	N/A	vH+,vA–
24	Histidine–trna cytoplasmic isoform x1	tr|F1RGD9|F1RGD9_PIG	4	1.36 × 10^−04^	3.553	72	Tetratricopeptide repeat protein 37	tr|F1RNV8|F1RNV8_PIG	3	N/A	vH+,vA–
25	fch domain only protein 2	tr|I3LSA6|I3LSA6_PIG	9	5.69 × 10^−03^	0.278	73	Isoleucine–trna cytoplasmic	tr|F1SUF6|F1SUF6_PIG	9	N/A	vH+,vA–
26	Denn domain‐containing protein 4c	tr|F1SNF8|F1SNF8_PIG	8	3.11 × 10^−03^	2.55	74	Collagen alpha‐1 chain	tr|F1S285|F1S285_PIG	8	N/A	vH+,vA–
27	Polyribonucleotide 5 ‐hydroxyl‐kinase clp1	tr|F2Z5N4|F2Z5N4_PIG	22	6.99 × 10^−03^	0.136	75	SH3 domain‐containing kinase‐binding protein 1 isoform x1	tr|K7GMW4|K7GMW4_PIG	4	N/A	vH+,vA–
28	Endophilin‐a2 isoform x1	tr|F1S7L8|F1S7L8_PIG	3	3.23 × 10^−03^	0.477	76	Rho gtpase‐activating protein 25	tr|F1SPM2|F1SPM2_PIG	2	N/A	vH+,vA–
29	Tyrosine‐protein phosphatase non‐receptor type 2 isoform x1	tr|I3L9Z5|I3L9Z5_PIG	40	8.58 × 10^−03^	0.419	77	Signal transducer and activator of transcription 6	tr|E1U8C5|E1U8C5_PIG	9	N/A	vH+,vA–
30	Unconventional myosin‐ixb	tr|F1S9U2|F1S9U2_PIG	23	5.24 × 10^−04^	14.095	78	Misshapen‐like kinase 1 isoform 1	tr|F1RFV9|F1RFV9_PIG	13	N/A	vH+,vA–
31	Ubiquitin‐fold modifier 1	tr|M3UZ42|M3UZ42_PIG	5	4.32 × 10^−03^	0.464	79	Coiled‐coil domain‐containing protein 61 isoform x1	tr|F1RM23|F1RM23_PIG	1	N/A	vH+,vA–
32	Trafficking protein particle complex subunit 8 isoform x2	tr|F1SAL9|F1SAL9_PIG	0	1.06 × 10^−05^	0.076	80	Histone ‐like	tr|I3LNZ2|I3LNZ2_PIG	7	N/A	vH–,vA+
33	h aca ribonucleoprotein complex subunit 4	tr|B7TJ11|B7TJ11_PIG	8	1.43 × 10^−03^	0.291	81	C5a anaphylatoxin chemotactic receptor	tr|I3LUE7|I3LUE7_PIG	12	N/A	vH–,vA+
34	Heterogeneous nuclear ribonucleoprotein u‐like protein 2	tr|I3LUR1|I3LUR1_PIG	2	1.36 × 10^−03^	0.457	82	Pyruvate dehydrogenase e1 component subunit mitochondrial	tr|F1SGH5|F1SGH5_PIG	7	N/A	vH–,vA+
35	3‐ketoacyl‐ peroxisomal isoform x1	tr|F1RRB7|F1RRB7_PIG	7	3.82 × 10^−03^	0.184	83	Ribosome biogenesis protein wdr12	tr|F1SHE8|F1SHE8_PIG	8	N/A	vH–,vA+
36	Hepatoma‐derived growth factor‐related protein 3	tr|F1RIB4|F1RIB4_PIG	2	3.32 × 10^−03^	0.423	84	Cleavage stimulation factor subunit 2 tau variant isoform x1	tr|F1SD01|F1SD01_PIG	3	N/A	vH–,vA+
37	Serine threonine‐protein kinase osr1	sp|Q863I2|OXSR1_PIG	12	9.51 × 10^−04^	0.385	85	Trifunctional enzyme subunit mitochondrial isoform x2	tr|F1SDN2|F1SDN2_PIG	9	N/A	vH–,vA+
38	Apoptosis‐associated speck‐like protein containing a card isoform 1	tr|K7GQI7|K7GQI7_PIG	22	5.20 × 10^−04^	0.345	86	Arf‐gap domain and fg repeat‐containing protein 2 isoform x1	tr|F1RMY4|F1RMY4_PIG	5	N/A	vH–,vA+
39	Trafficking protein particle complex subunit 8	tr|F1SAL8|F1SAL8_PIG	10	2.98 × 10^−03^	3.623	87	Sam and sh3 domain‐containing protein 3	tr|F1RTH8|F1RTH8_PIG	14	N/A	vH–,vA+
40	Squamous cell carcinoma antigen recognized by t‐cells 3 isoform x2	tr|F1RGA7|F1RGA7_PIG	8	5.52 × 10^−03^	0.34	88	Actin‐like protein 6a	tr|F1SGC8|F1SGC8_PIG	13	N/A	vH–,vA+
41	Enoyl‐ delta isomerase mitochondrial	tr|A9 × 3T3|A9 × 3T3_PIG	7	1.17 × 10^−03^	0.493	89	TBC1 domain family member 10b	tr|F1RG61|F1RG61_PIG	2	N/A	vH–,vA+
42	wd repeat‐containing protein 7 isoform x2	tr|F1S1 × 8|F1S1 × 8_PIG	1	7.90 × 10^−03^	2.052	90	Deubiquitinating protein vcip135	tr|F1RTZ5|F1RTZ5_PIG	3	N/A	vH–,vA+
43	Dipeptidyl peptidase 9	tr|M3VH83|M3VH83_PIG	0	6.78 × 10^−06^	37.804	91	GNAS complex locus	tr|A5GFU0|A5GFU0_PIG	38	N/A	vH–,vA+
44	Glycerol‐3‐phosphate dehydrogenase 1‐like protein	tr|I3LLU0|I3LLU0_PIG	20	9.36 × 10^−03^	0.461	92	Protein red‐like	tr|F1RGE2|F1RGE2_PIG	9	N/A	vH–,vA+
45	Serine arginine repetitive matrix protein 2	tr|I3LCW3|I3LCW3_PIG	6	2.18 × 10^−03^	0.489	93	Signal‐induced proliferation‐associated protein 1	tr|F1RRK2|F1RRK2_PIG	17	N/A	vH–,vA+
46	Protein mago nashi homolog	tr|F1S766|F1S766_PIG	14	3.89 × 10^−03^	0.487	94	Protein virilizer homolog	tr|F1RY51|F1RY51_PIG	0	N/A	vH–,vA+
47	Camp‐dependent protein kinase catalytic subunit alpha isoform x1	sp|P36887|KAPCA_PIG	31	1.25 × 10^−03^	2.053	95	GDH 6pgl endoplasmic bifunctional protein	tr|K9IVK1|K9IVK1_PIG	6	N/A	vH–,vA+
48	Interferon‐induced transmembrane protein 3	tr|E7EAX3|E7EAX3_PIG	2	8.57 × 10^−03^	2.862						

Uniprot accession and Seq. description are collected by the blast2go (http://www.blast2go.com). And the names of the proteins are identified by the accession number from UniProt. #GOs means the quantity of GO annotation, and the HP‐PRRSV/AP‐PRRSV stands for the ratio between the quantity of expression between HP‐PRRSV and AP‐PRRSV group.

### 3.2 Subcellular and Functional Characterization and Bioinformatics of Differently Expressed Proteins between the HP‐PRRSV and AP‐PRRSV Groups

**Figure 4:**
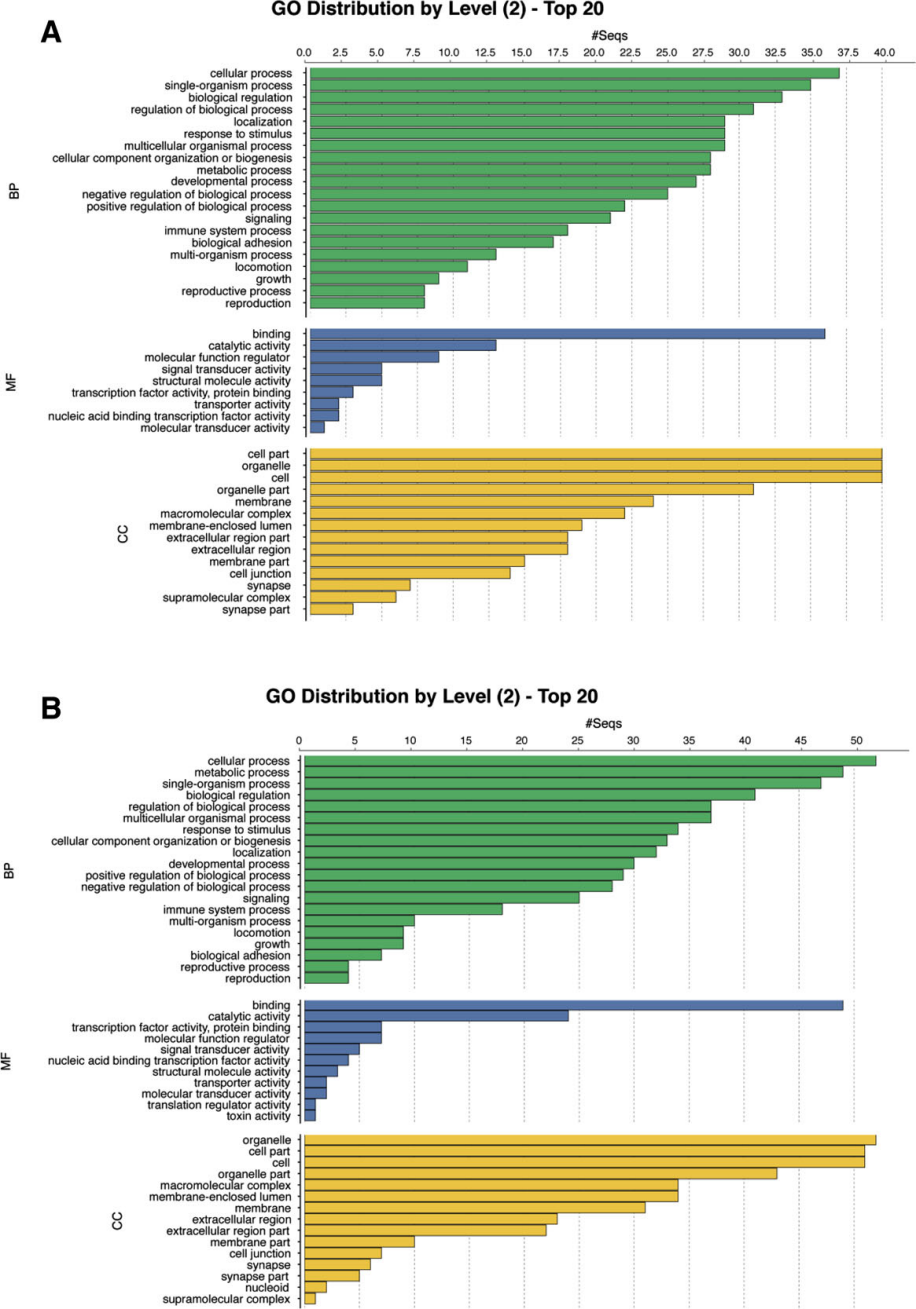
GO categories of differentially expressed proteins between the HP‐PRRSV‐infected A) and AP‐PRRSV‐infected. B) BP, biological process GO categories; MF, molecular function GO categories; CC, cellular component GO categories.

To extend the molecular characterization of quantitative differences and only‐one‐exists groups, UniProt and GO databases were used to characterize information about biological processes (BPs), molecular functions (MF), and cellular components (CC). BPs of H (vH/vA >2, including 41 proteins) and A (vH/vA < 0.5, including 54 proteins) groups are in Figures 4A and B BP. In group H, GO annotations were primarily distributed in response to stimulus (70.7%), metabolic process (68.3%), and immune system process (43.9%). Ratios were 63.0% response to stimulus, 90.7% metabolic process, and 33.3% immune system process in group A. The most significant difference in BP between PAMs infected with HP‐PRRSV and AP‐PRRSV was seen for metabolic process, which may be the major reason for the large differences among animals challenged with different virulence of PRRSV.

Molecular function categories of H group and A group were shown in Figure 4A,B MF. Most different molecular functions were classified as binding (87.8 and 90.7%) and catalytic activity (31.7 and 44.4%). Binding of H group included enzyme binding (31.7%), nucleic acid binding (31.7%) and protein complex binding (26.8%), while group A mainly involved nucleic acid binding (31.5%), nucleoside phosphate binding (27.8%) and nucleotide binding (27.8%) from the analysis of GO distribution by level 4. Enzyme code distribution suggested there were five transferases (12.2%), one hydrolase (2.4%), one lyase (2.4%), and two ligases (4.9%) detected in group H. while four oxidoreductases (1.9%), five transferases (9.3%), five hydrolases (9.3%), three lyases (5.6%), and three isomerases (5.6%) in group A.

CC categories were illustrated in Figure 4A B CC. Ninety‐five detected differentially expressed proteins were annotated and categorized to CCs of macromolecular complex (58.9%), membrane (57.9%), membrane‐enclosed lumen (57.9%), and extracellular region (44.2%). Because of technological problems, we were unable to conclude that all the detected proteins were indeed MPs. However, 26 were confirmed based on the annotation of UniProt database and there also existed proteins partially anchored on the membrane or binding with the MPs.

### 3.3 Validation of Differentially Expressed MPs by Western Blots

**Figure 5:**
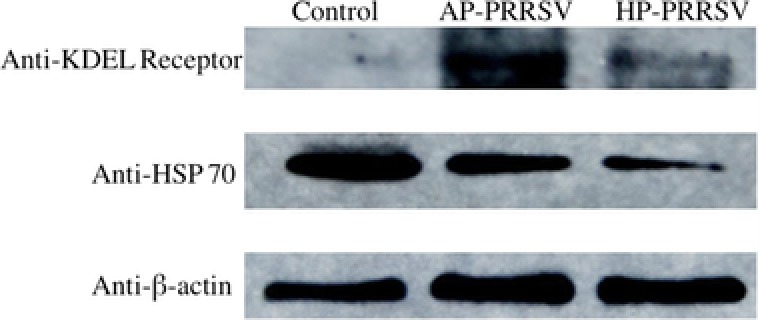
Verification of LFQP results by Western blot.

To verify the differentially expressed proteins via LC‐MS/MS for LFQP, Western blots were conducted for two proteins partially located on membranes. Expression of HSP70 and the KDEL receptor from cell lysates of vHuN4‐infected and vHuN4‐F112‐infected PAMs, and DMEM‐inoculated PAMs were tested with antibodies to the proteins. LFQP showed that the ratios between vHuN4‐infected and vHuN4‐F112‐infected PAMs reached 0.67 and 0.51, respectively. Western blots confirmed LFQP results (Figure 5).

### 3.4 VCL Protein Inhibits Virus Replication

**Figure 6:**
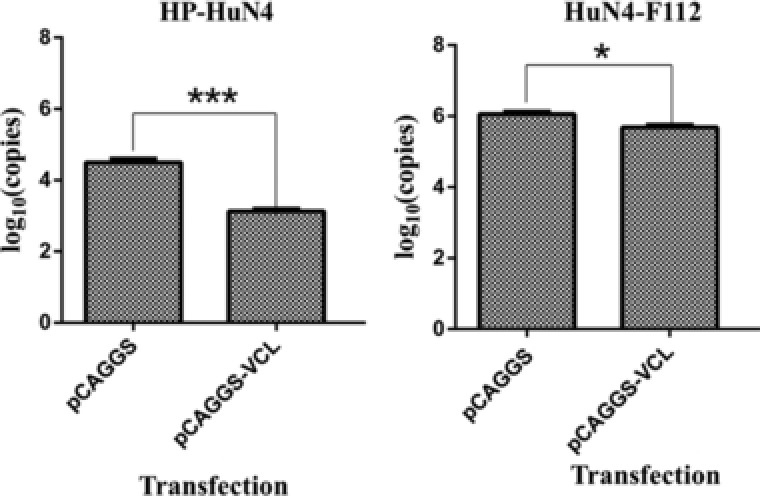
Virus replication detected for HP‐HuN4 or its attenuated HuN4‐F112 stain after transient expression of VCL protein. *** indicated extremely significant difference. * indicated significant difference. pCAGGS transfection indicated transfected using empty vector.

To examine whether the differentially expressed proteins detected affects virus infection, the VCL transient overexpression vector was transfected into MARC‐145 cells, followed by the HP HuN4 or its attenuated strain infection. The PRRSV‐specific RT‐qPCR results showed that VCL protein could inhibit both viruses, especially for HP‐HuN4 strain. Compared with the empty vector control, the virus titer of HP‐HuN4 dramatically decreased, while viral titer of attenuated strain mildly decreased, as shown in Figure 6.

## 4 Discussion

**Figure 7:**
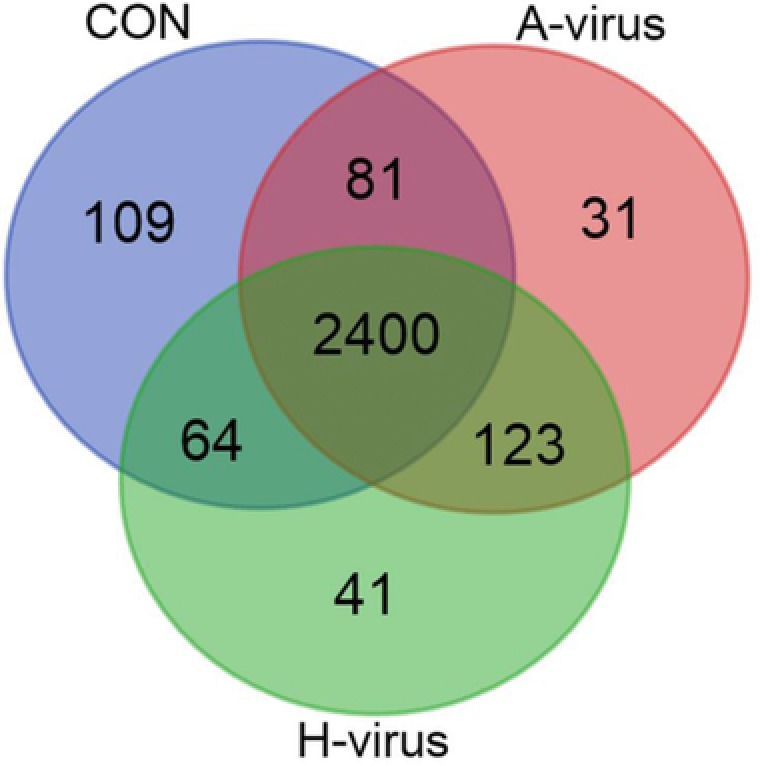
Venn diagrams of protein quantities in different groups by LFQP. Control, AP‐PRRSV, and HP‐PRRSV groups are represented by different colors. Overlapping areas indicate proteins shared between two or three groups. Numbers within individual circles, differentially expressed and shared proteins. Number 2400 was common to all three groups. Number 31 was detected only in the AP‐PRRSV. Number 41 was detected only in HP‐PRRSV. Number 109 was detected only in control. Number 81 was detected in control and AP‐PRRSV, but not HP‐PRRSV. Number 64 was detected in control and HP‐PRRSV, but not AP‐PRRSV. Number 123 was detected in AP‐PRRSV and HP‐PRRSV, but not control.

In this study, LFQP of MPs of HP‐PRRSV‐, AP‐PRRSV‐infected PAMs and the control was performed. Important information about target proteins related to virus infection was obtained. The HP‐PRRSV strain vHuN4 and its derivative, the serially cell‐passaged attenuated strain vHuN4‐F112,^29^ revealed different infection mechanisms in PAMs. A total of 2849 proteins were identified among the control, AP‐PRRSV‐infected, and HP‐PRRSV‐infected PAMs. Of these, 2400 were detected in all three groups (Figure 7). We focused on 95 differentially expressed proteins of AP‐PRRSV‐ and HP‐PRRSV‐infected PAMs; among these, 43 were detected in only one group.

### 4.1 Identification and Potential Function of MPs in Immunology and Inflammation

**Figure 8:**
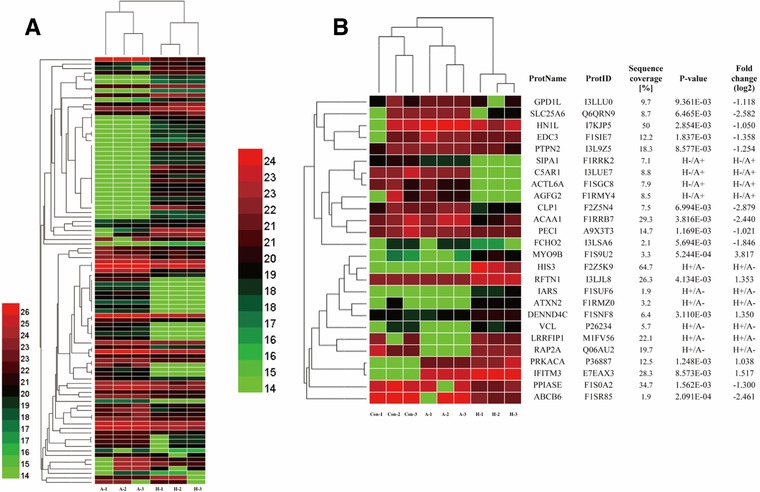
Heatmaps of differently expressed proteins. A) Heatmap of 95 differentially expressed proteins. B) Heatmap of 26 differently expressed membrane proteins. Values of protein expression loaded three times are displayed. Data were clustered by hierarchical average linage.^88^ Color depth indicates expression abundance. Green, no, or low expression; red, high expression. Con‐1, Con‐2, and Con‐3 are control samples. A‐1, A‐2, and A3 are AP‐PRRSV samples. H‐1, H‐2, and H‐3 are HP‐PRRSV samples.

PRRSV has been a threat to the global pig economy for several years because of its persistent infection, immune escape, and high mortality from inflammation and high fever.^30^ The attenuated PRRSV vHuN4‐F112 vaccine strain attenuated from HP‐PRRSV vHuN4 by serial passages, which is now used in China.^31^ We analyzed MPs to identify factors associated with immunological effects and determine differences between HP‐PRRSV and AP‐PRRSV. MPs classified based on GO analysis are in Table 3. A heatmap based on the UniProt database was constructed to comprehend the functions and BPs of differently expressed proteins (Figure 8B), which revealed clustering and abundance of the 26 detected proteins that existed confidentially on the membrane based on UniProt database annotation.

**Table 3:** Statistics analysis of proteins that existed definitely on the membrane

UniProt accession	Description	Cellular component	Molecular function	Biological process	HP/AP‐PRRSV
**Quantitative Difference**					
tr|F1SIE7|F1SIE7_PIG	Enhancer of mRNA‐decapping protein 3	Cytoplasmic mRNA processing bodymembrane	RNA binding; identical protein binding	NA	0.390
tr|F1SR85|F1SR85_PIG	Low quality protein: ATP‐binding cassette sub‐family b member mitochondrial	Endosome; endoplasmic reticulum; Golgi apparatus; plasma membrane; integral component of mitochondrial outer membrane; extracellular vesicular exosome	ATP binding; heme‐transporting ATPase activity; efflux transmembrane transporter activity; heme binding; ATP catabolic process	Porphyrin‐containing compound biosynthetic process; brain development; heme transport; skin development; transmembrane transport	0.182
tr|I7KJP5|I7KJP5_PIG	Hematological and neurological expressed 1‐like protein	Cytoplasm; plasma membrane	NA	NA	0.483
tr|I3LJL8|I3LJL8_PIG	Raftlin‐ partial	Endosome; plasma membrane; protein complex; membrane raft; extracellular vesicular exosome	Double‐stranded RNA binding	Membrane raft assembly; T cell antigen processing and presentation; protein transport into membrane raft; IL‐17 production; dsRNA transport; toll‐like receptor 3 signaling pathway; growth; response to exogenous dsRNA; T cell receptor signaling pathway; B cell receptor signaling pathway	2.554
sp|Q6QRN9|ADT3_PIG	ADP ATP translocase 3	Nucleus; mitochondrial inner membrane presequence translocase complex; integral component of membrane	ATADP antiporter activity; protein binding	Energy reserve metabolic process; protein targeting to mitochondrion; apoptotic process; ADP transport; ATP transport; viral life cycle; cellular protein metabolic process; small molecule metabolic process; active induction of host immune response by virus; regulation of insulin secretion; transmembrane transport	0.167
tr|F1S0A2|F1S0A2_PIG	Peptidyl‐prolyl cis‐trans isomerase b	Nucleus; endoplasmic reticulum; membrane; macromolecular complex; extracellular vesicular exosome	Peptidyl‐prolyl cis‐trans isomerase activity; protein complex binding poly(A) RNA binding	Protein peptidyl‐prolyl isomerization; positive regulation of multicellular organism growth; protein stabilization; bone development; chaperone‐mediated protein folding	0.406
tr|I3LSA6|I3LSA6_PIG	fch domain only protein 2	Plasma membrane; coated pit; clathrin‐coated vesicle	Phosphatidylserine binding; phosphatidylinositol‐4,5‐bisphosphate binding	Membrane invagination; clathrin coat assembly; clathrin‐mediated endocytosis; protein localization to plasma membrane	0.278
tr|F1SNF8|F1SNF8_PIG	Denn domain‐containing protein 4c	Cytosol; plasma membrane; retromer complex; insulin‐responsive compartment	Rab guanyl‐nucleotide exchange factor activity	Positive regulation of Rab GTPase activity; cellular response to insulin stimulus; protein localization to plasma membrane	2.550
tr|F2Z5N4|F2Z5N4_PIG	Polyribonucleotide 5 ‐hydroxyl‐kinase clp1	tRNA‐intron endonuclease complex; collagen trimer; mRNA cleavage factor complex; integral component of membrane; extracellular vesicular exosome	ATP binding; signaling pattern recognition receptor activity; low‐density lipoprotein particle binding; carbohydrate binding; ATP‐dependent polydeoxyribonucleotide 5'‐hydroxyl‐kinase activity; metal ion binding; ATP‐dependent polyribonucleotide 5'‐hydroxyl‐kinase activity	Pattern recognition receptor signaling pathway; mRNA polyadenylation; mRNA cleavage; tRNA splicing, via endonucleolytic cleavage and ligation; phagocytosis, recognition; immune response; phosphorylation; cerebellar cortex development; targeting of mRNA for destruction involved in RNA interference; siRNA loading onto RISC involved in RNA interference	0.136
tr|I3L9Z5|I3L9Z5_PIG	Tyrosine‐protein phosphatase non‐receptor type 2 isoform x1	Nucleus; endoplasmic reticulum; endoplasmic reticulum‐golgi intermediate compartment; plasma membrane;	Protein tyrosine phosphatase activity; integrin binding; protein kinase binding; syntaxin binding; receptor tyrosine kinase binding; negative regulation of cell proliferation;	Insulin receptor signaling pathway; negative regulation of tumor necrosis factor‐mediated signaling pathway; negative regulation of lipid storage; B cell differentiation; T cell differentiation; erythrocyte differentiation; peptidyl‐tyrosine dephosphorylation; negative regulation of epidermal growth factor receptor signaling pathway; negative regulation of tyrosine phosphorylation of Stat1 protein; negative regulation of tyrosine phosphorylation of Stat3 protein; negative regulation of tyrosine phosphorylation of Stat5 protein; negative regulation of tyrosine phosphorylation of Stat6 protein; glucose homeostasis; negative regulation of macrophage differentiation; positive regulation of gluconeogenesis; negative regulation of insulin receptor signaling pathway; negative regulation of inflammatory response; negative regulation of T cell receptor signaling pathway; negative regulation of chemotaxis; negative regulation of IFN‐gamma‐mediated signaling pathway; negative regulation of type I IFN‐mediated signaling pathway; negative regulation of IL‐6‐mediated signaling pathway; negative regulation of ERK1 and ERK2 cascade; regulation of hepatocyte growth factor receptor signaling pathway; negative regulation of IL‐2‐mediated signaling pathway; negative regulation of prolactin signaling pathway; negative regulation of IL‐4‐mediated signaling pathway; negative regulation of macrophage colony‐stimulating factor signaling pathway; negative regulation of positive thymic T cell selection; negative regulation of platelet‐derived growth factor receptor‐beta signaling pathway	0.419
tr|F1S9U2|F1S9U2_PIG	Unconventional myosin‐ixb	ruffle; cell cortex; membrane; myosin complex; lamellipodium; filamentous actin; filopodium tip; perinuclear region of cytoplasm;	Microfilament motor activity; actin binding; Rho GTPase activator activity; calmodulin binding; ATP binding; ATPase activity; ADP binding; metal ion binding;	Monocyte chemotaxis; ATP catabolic process; Rho protein signal transduction; establishment of cell polarity; positive regulation of Rho GTPase activity; macrophage chemotaxis; lamellipodium morphogenesis	14.095
tr|F1RRB7|F1RRB7_PIG	3‐ketoacyl‐ peroxisomal isoform x1	Peroxisome; membrane;	Palmitoyl‐CoA oxidase activity; transferase activity, transferring acyl groups other than amino‐acyl groups;	Very long‐chain fatty acid metabolic process; fatty acid beta‐oxidation; bile acid metabolic process	0.184
tr|A9 × 3T3|A9 × 3T3_PIG	Enoyl‐ delta isomerase mitochondrial	Nucleus; mitochondrion; membrane	Fatty‐acyl‐CoA binding; receptor binding; isomerase activity	Fatty acid catabolic process	0.493
tr|I3LLU0|I3LLU0_PIG	Glycerol‐3‐phosphate dehydrogenase 1‐like protein	Plasma membrane; glycerol‐3‐phosphate dehydrogenase complex; extracellular vesicular exosome	Glycerol‐3‐phosphate dehydrogenase [NAD+] activity; sodium channel regulator activity; protein homodimerization activity; ion channel binding; NAD binding	Regulation of heart rate; carbohydrate metabolic process; NADH metabolic process; positive regulation of sodium ion transport; negative regulation of peptidyl‐serine phosphorylation; glycerol‐3‐phosphate catabolic process; oxidation‐reduction process; regulation of ventricular cardiac muscle cell membrane depolarization; ventricular cardiac muscle cell action potential; negative regulation of protein kinase C signaling; positive regulation of protein localization to cell surface; regulation of sodium ion transmembrane transporter activity	0.461
sp|P36887|KAPCA_PIG	cAMP‐dependent protein kinase catalytic subunit alpha isoform x1	Nucleus; mitochondrion; centrosome; plasma membrane; AMP‐activated protein kinase complex; neuromuscular junction; extracellular vesicular exosome; sperm midpiece; ciliary base	cAMP‐dependent protein kinase activity; protein serine/threonine/tyrosine kinase activity; ATP binding; protein kinase binding; ubiquitin protein ligase binding; protein kinase A regulatory subunit binding	Mesoderm formation; neural tube closure; peptidyl‐serine phosphorylation; peptidyl‐threonine phosphorylation; regulation of osteoblast differentiation; protein autophosphorylation; positive regulation of protein export from nucleus; sperm capacitation; regulation of synaptic transmission; regulation of proteasomal protein catabolic process; regulation of protein processing; positive regulation of cell cycle arrest; cellular response to glucose stimulus; cellular response to parathyroid hormone stimulus; negative regulation of smoothened signaling pathway involved in dorsal/ventral neural tube patterning; regulation of tight junction assembly	2.053
tr|E7EAX3|E7EAX3_PIG	IFN‐induced transmembrane protein 3	Integral component of membrane;	NA	Response to biotic stimulus	2.862
**“Only one exists"**					
sp|Q06AU2|RAP2A_PIG	RAS‐related protein rap‐2b	Cytosol; plasma membrane; midbody; membrane raft; recycling endosome membrane; extracellular vesicular exosome	GTPase activity; GTP binding; GDP binding; protein domain specific binding	GTP catabolic process; negative regulation of cell migration; actin cytoskeleton reorganization; positive regulation of protein autophosphorylation; Rap protein signal transduction; cellular protein localization; regulation of JNK cascade; regulation of dendrite morphogenesis; regulation of protein tyrosine kinase activity; platelet aggregation	vA– vH+
tr|F2Z5K9|F2Z5K9_PIG	Histone ‐like	Nuclear chromosome; nucleosome; nucleoplasm; membrane; extracellular vesicular exosome	DNA binding; chromatin binding; protein heterodimerization activity	Negative regulation of transcription from RNA polymerase II promoter; chromatin silencing at rDNA; DNA replication‐dependent nucleosome assembly; blood coagulation; gene expression; DNA methylation on cytosine; cellular response to stress; regulation of gene silencing	vA– vH+
tr|M1FV56|M1FV56_PIG	Leucine‐rich repeat interacting protein‐1	Cytoplasm; plasma membrane	DNA binding; protein homodimerization activity	NA	vA– vH+
tr|F1RMZ0|F1RMZ0_PIG	Ataxin‐2 isoform x2	Nucleus; trans‐Golgi network; polysome; cytoplasmic stress granule; membrane; perinuclear region of cytoplasm	Epidermal growth factor receptor binding; protein C‐terminus binding; poly(A) RNA binding	Negative regulation of receptor internalization; cerebellar Purkinje cell differentiation; cytoplasmic mRNA processing body assembly; stress granule assembly; negative regulation of multicellular organism growth; neuron projection morphogenesis; homeostasis of number of cells; neuromuscular process	vA– vH+
sp|P26234|VINC_PIG	Vinculin isoform x1	Cytosol; plasma membrane; fascia adherens; focal adhesion; actin cytoskeleton; costamere; protein complex; extracellular vesicular exosome	Dystroglycan binding; actin binding; structural molecule activity; beta‐catenin binding; alpha‐catenin binding; cadherin binding	Morphogenesis of an epithelium; platelet degranulation; movement of cell or subcellular component; muscle contraction; cell‐matrix adhesion; lamellipodium assembly; negative regulation of cell migration; adherens junction assembly; protein localization to cell surface; apical junction assembly; platelet aggregation; epithelial cell–cell adhesion	vA– vH+
tr|F1SUF6|F1SUF6_PIG	Isoleucine–trna cytoplasmic	Cytosol; membrane; extracellular vesicular exosome	Aminoacyl‐tRNA editing activity; isoleucine‐tRNA ligase activity; ATP binding	Osteoblast differentiation; isoleucyl‐tRNA aminoacylation; regulation of translational fidelity	vA– vH+
tr|I3LUE7|I3LUE7_PIG	C5a anaphylatoxin chemotactic receptor	Integral component of membrane; basolateral plasma membrane; apical part of cell	Complement component C5a receptor activity; C5a anaphylatoxin receptor activity	Neutrophil chemotaxis; response to peptidoglycan; complement component C5a signaling pathway; mRNA transcription from RNA polymerase II promoter; positive regulation of epithelial cell proliferation; defense response to gram‐positive bacterium; positive regulation of ERK1 and ERK2 cascade	vA+ vH–
tr|F1RMY4|F1RMY4_PIG	ARF‐gap domain and fg repeat‐containing protein 2 isoform x1	Membrane	ARF GTPase activator activity; zinc ion binding	Regulation of ARF GTPase activity; positive regulation of GTPase activity	vA+ vH–
tr|F1SGC8|F1SGC8_PIG	Actin‐like protein 6a	Plasma membrane; SWI/SNF complex; Ino80 complex; NuA4 histone acetyltransferase complex; npBAF complex	RNA polymerase II core promoter proximal region sequence‐specific DNA binding; RNA polymerase II distal enhancer sequence‐specific DNA binding; nucleosomal DNA binding	Neural retina development; nervous system development; ATP‐dependent chromatin remodeling; histone H4 acetylation; histone H2A acetylation	vA+ vH–
tr|F1RRK2|F1RRK2_PIG	Signal‐induced proliferation‐associated protein 1	Nucleolus; Golgi apparatus; cytosol; plasma membrane; transport vesicle; protein complex; perinuclear region of cytoplasm	Protein C‐terminus binding; Rap GTPase activator activity	Cytoskeleton organization; negative regulation of cell adhesion; cell proliferation; negative regulation of cell growth; positive regulation of Rap GTPase activity; intracellular signal transduction; cellular response to water deprivation; negative regulation of cell cycle	vA+ vH–

Cellular component, molecular function and biological process are obtained from the UniProt database.

We first focused on proteins associated with immunology and inflammation with higher abundance in AP‐PRRSV‐infected PAMs. PTPN2 (vH/vA = 0.419), a member of the protein tyrosine phosphatase family, functions as signaling molecule that regulates cellular processes related to the Jak‐STAT, IL‐3, IL‐5, and granulocyte‐macrophage colony‐stimulating factor (GM‐CSF) signaling pathways; dephosphorylation of nonreceptor kinases including JAK1, JAK3, STAT3, and STAT6; and negative regulation of IL‐2‐, IL‐4, IL‐6, and IFN‐mediated signaling. PTPN2 also functions in the response to inflammation via NF‐κB.^32^, ^33^, ^34^ SIPA1 (H‐/A+) is a mitogen‐induced GTPase activating protein for Ras‐related regulatory proteins. It was related to G‐protein signaling and blood‐brain barrier and immune cell transmigration: VCAM01/CD106 (cluster of differentiation) signaling pathways.^35^, ^36^ C5AR1 (H–/A+) is the receptor for the chemotactic and inflammatory peptide anaphylatoxin C5a, which stimulates chemotaxis, granule enzyme release, intracellular calcium release, and superoxide anion production, participates in the innate and adaptive immune responses to the lectin‐induced complement pathway.^37^, ^38^, ^39^, ^40^, ^41^, ^42^ Polyribonucleotide 5′‐hydroxyl‐kinase Clp1 (CLP1) (vH/vA = 0.136) mainly acts as a kinase that binds ATP hosting 5′‐hydroxyl‐kinase activity, and functions in mRNA cleavage and in siRNA loading onto the RNA‐induced silencing complex involved in RNA interference and its destruction. The kinase hClp1 phosphorylates and licenses synthetic siRNAs to assemble into an RNA‐induced silencing complex for cleavage of target RNA.^43^ Expression of these molecules was higher in the AP‐PRRSV‐infected group and had a similar level to the control group. Therefore, we concluded that they might be related to the immune escape of HP‐PRRSV and that the attenuated vaccine strain would not have side effects on these immune factors expression in the host cell, and these proteins above would contribute to the resistance of PRRSV.

Some proteins related to immunology and inflammation had higher expression in HP‐PRRSV‐infected cells. Raftlin (vH/vA = 2.554) protein is pivotal for maintenance of lipid rafts and may be involved in regulation of B‐cell antigen receptor‐mediated signaling. Raftlin promotes binding of double‐stranded RNA, activations of B cell receptors and toll‐like receptor 3 signaling pathways, and is involved in IL‐17 production to release pro‐inflammatory cytokines. Hence, the higher abundance of raftlin in the HP‐PRRSV group compared to the AP‐PRRSV and control groups may explain the more severe inflammation triggered by HP‐PRRSV.^44^, ^45^ IARS (H+/A‐), Isoleucyl‐tRNA synthetase is a target of autoantibodies in autoimmune diseases.^46^, ^47^ VCL (H+/A–), Vinculin is a cytoskeletal protein associated with cell–cell and cell–matrix junctions, and is related to the IL‐3, IL‐5, and GM‐CSF signaling pathways.^48^ RAP2A (H+/A‐) is a member of the Ras oncogene family, small GTP‐binding protein, of which active form interacts with several effectors^49^, ^50^, ^51^ related to the ERK and G protein signaling pathways.^52^, ^53^ It was reported that PRRSV could induces prostaglandin E2 production through cyclooxygenase 1 and is related to ERK signaling.^54^ The differential expression of RAP2A may provide information for future research on the interaction between PRRSV and the ERK signaling pathway. The abundance of RAP2A was lower in AP‐PRRSV‐infected PAMs than in HP‐PRRSV‐infected PAMs and control cells, which had similar levels. Thus, we hypothesized that RAP2A was associated with immunization with the attenuated vaccine. IFN‐induced transmembrane protein 3, IFITM3 (vH/vA = 2.861), responds to biotic stimulus and had the highest expression levels among these proteins in the HP‐PRRSV group, as compared to the proteins identified in the AP‐PRRSV group but it was not detected in the control group. In humans, this protein negatively regulates the entry of multiple viruses, including influenza A virus, SARS coronavirus, Marburg virus (MARV), Ebola virus, Dengue virus, West Nile virus (WNV), human immunodeficiency virus (HIV) type 1, and vesicular stomatitis virus (VSV), into host cells.^55^, ^56^ It could inhibit hemagglutinin protein –mediated entry of influenza virus, GP1, two‐mediated viral entry of Marburg virus and Ebola virus, S protein‐mediated viral entry of SARS coronavirus, and G protein‐mediated viral entry of VSV, thereby playing a critical role in the structural stability and function of vacuolar ATPase (v‐ATPase). Establishing physical contact with the v‐ATPase of endosomes is critical for proper clathrin localization and is required for v‐ATPase to lower the pH in phagocytic endosomes, thus establishing an antiviral state.^57^ High expression of both HP‐PRRSV and AP‐PRRSV suggests that IFITM3 may help inhibit PRRSV entry and promote resistance to HP‐PRRSV infection. Peptidyl‐prolyl cis‐trans isomerase B (PPIB, vH/vA = 0.406) has peptidyl‐prolyl cis‐trans isomerase activity and is involved in protein folding and protein peptidyl‐prolyl isomerization, which can accelerate protein folding.^58^ PPIB is positively regulated by viral genome replication, is involved with the viral processes of hepatitis C virus (HCV), and interacts with and stimulates the RNA‐binding activity of HCV NS5B. PPIB is critical for efficient replication of the HCV genome.^59^ Compared with the control group, PPIB was downregulated in the HP‐PRRSV and AP‐PRRSV groups. The abundance of PPIB was lower in the HP‐PRRSV group than the AP‐PRRSV group, suggesting an association with PRRSV replication.

### 4.2 Potential Different Approaches Used by Both PRRSVs

**Figure 9:**
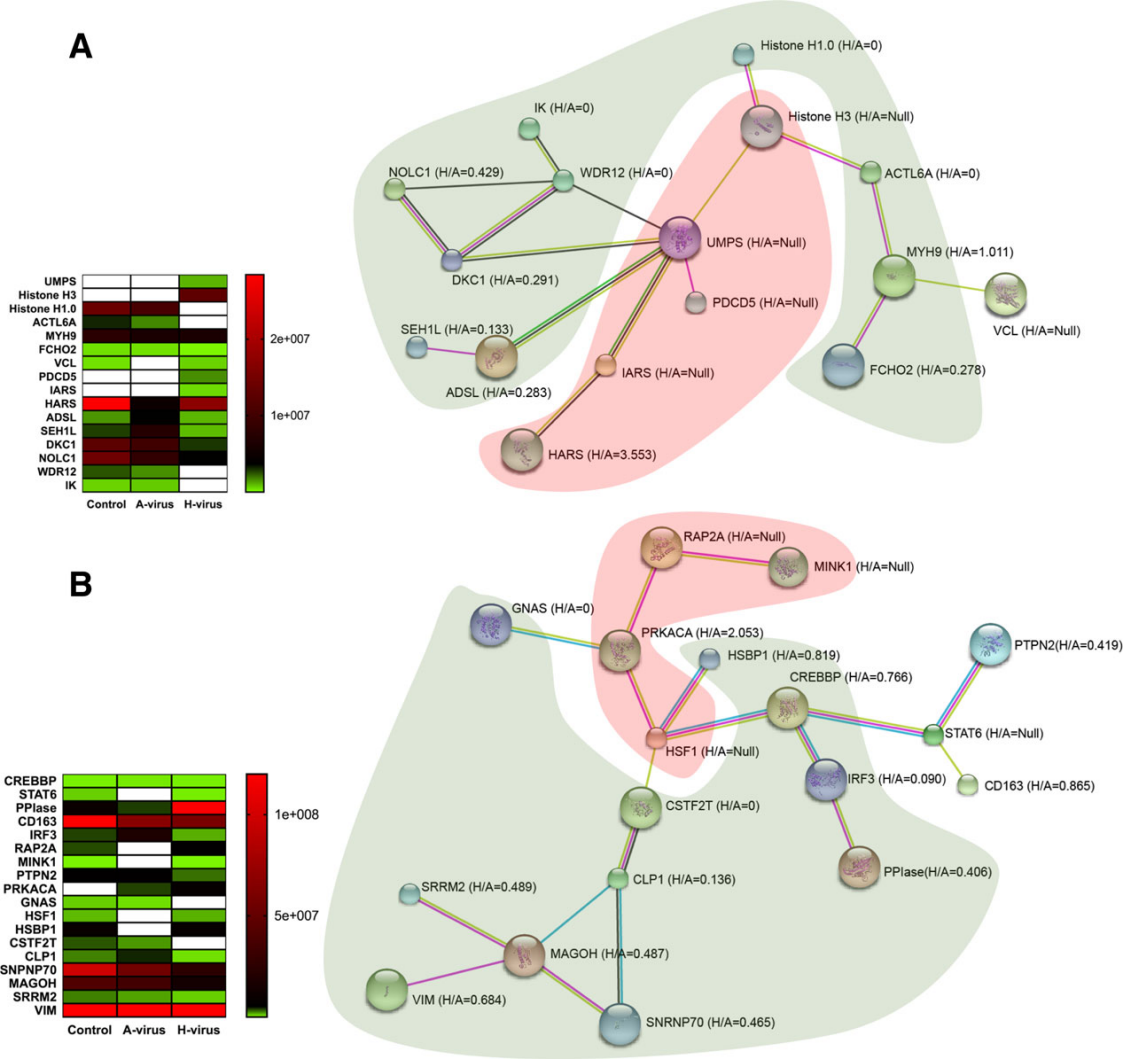
Protein‐protein interaction network determined by String software showing interactions among differentially expressed proteins between two virulent groups with CD163, VIM, and MYH9 added. Red, protein abundance higher in HP‐PRRSV than AP‐PRRSV; green, protein abundance in HP‐PRRSV lower than in AP‐PRRSV. Line colors represent type of evidence for association: green, neighborhood; red, fusion; purple, experimental; light blue, database; black, expression; blue, co‐occurrence; yellow, text mining. Gene abbreviations are shown. vH, protein abundance in HP‐PRRSV; vA protein abundance in AP‐PRRSV. vH/vA = 0, detected only in AP‐PRRSV; vH/vA = null, detected only in HP‐PRRSV.

String network analysis was used to elucidate interactions among differentially expressed proteins. The essential factors and receptors involved in the entry of PRRSV are reportedly CD163 (Q2VL90), nmHC II‐A (MYH9, F1SKJ1), sialoadhesin (SN, A7LCJ3), CD151 (http://F1RYZ1), and vimentin (VIM) (P02543).^60^, ^61^, ^62^, ^63^, ^64^, ^65^ We assessed the involvement of these MPs together with proteins detected by string analysis on viral entry. Receptor proteins involved in PRRSV entry are in Figure 9 CD163 (WC1), nmHC II‐A (MYH9), and VIM were related to a series of pathway‐like regions. Detected virus entry‐related receptors and guiding pathway‐like regions participated in interactions of AP‐PRRSV, HP‐PRRSV, and a combination of both (Figure. 9).

To better understand the characteristics of pathway‐like regions, the GenomRNAi database was analyzed to annotate detected proteins.^66^ Regions containing proteins with higher abundance in HP‐PRRSV‐infected PAMs are green and in AP‐PRRSV‐infected PAMs are red (Figure 9A). A search of the GenomRNAi database determined that linked proteins IK (IK cytokine), WD repeat domain 12, dyskerin pseudouridine synthase 1, and adenylosuccinate lyase (ADSL) in the green region of Figure 9A shared similar annotations and functioned to decrease expression of NF‐κB or IL‐8,^67^ which are related to inflammation.^44^, ^45^ Hence, further analysis of these linked proteins may help explain differential mechanisms between HP‐PRRSV and AP‐PRRSV infection. Other proteins in the green‐colored region of 9A are reported to influence virus infection. Nucleolar and coiled‐body phosphoprotein 1 increases Sindbis virus infection^68^ and ADSL increases human papilloma virus 16‐GFP infection.^69^ The lower abundance of nucleolar and coiled‐body phosphoprotein and ADSL in the HP‐PRRSV‐infected group may be related to the host immune response repression of PRRSV and may be used by HP‐PRRSV during infection to self‐upregulate. The red region of Figure 9A contains some linked proteins with some information. Uridine monophosphate synthetase decreases HIV‐1 infection,^70^ while programmed cell death 5 decreases HCV replication,^71^ both downregulate NF‐κB expression.^67^ The lower abundance of these proteins in the HP‐PRRSV group suggested that HP‐PRRSV escaped inhibition through an unknown mechanism. Myosin‐9 (MYH9) appears to function in cytokinesis, cell shape, and specialized functions such as secretion and capping.^72^ MYH9 is an important factor for PRRSV infection and interacts with GP5 of PRRSV,^65^ although the underlying mechanisms remain unclear because of limited research. As shown by our results, MYH9 was related to VCL in AP‐PRRSV and ACTL6 in HP‐PRRSV. VCL is an actin filament (F‐actin)‐binding protein involved in cell–matrix adhesion and cell–cell adhesion in humans, regulation of cell‐surface E‐cadherin expression, and potentiation of mechanosensing, and may be important in cell morphology and locomotion by promoting binding with actin, alpha‐catenin, cadherin, dystroglycan, and ubiquitin protein ligase.^73^ In HIV research, transient overexpression of VCL reduced the susceptibility of human cells to infection with HIV‐1 and negatively affected paxillin phosphorylation and limited retroviral infection.^74^ Just like HIV‐1, in our study, it was demonstrated that over expression of VCL could inhibit the replication of both HP‐PRRSV and the attenuated PRRSV in the mRNA level. There were obvious differences in the inhibiting ability for HP‐PRRSV and its attenuated strain. ACTL6A, which is an actin‐like protein 6A, is involved in transcriptional activation and repression of select genes by chromatin remodeling (alteration of DNA‐nucleosome topology) and mainly functions in chromatin binding and transcription coactivator activities.^75^ We found that MYH9 may regulate AP‐PRRSV and HP‐PRRSV infection via different pathways. Through Super Pathways annotation (http://www.genecards.org), VCL and ACTL6A were identified as participants in the IL‐3, IL‐5, GM‐CSF, and TNF‐α/NF‐κB signaling pathways, where they may help with differential mechanisms of PRRSV infection with different virulence.

Using the GenomRNAi database, some linked proteins were found to be related to viruses or inflammation (Figure 9B). In the green region, GNAS complex locus (GNAS) increased human papilloma virus 16‐GFP,^69^ and serine/arginine repetitive matrix 2 decreased HCV infection, influenza A replication and viral numbers, and IL‐8 expression,^76^, ^77^ Small nuclear ribonucleoprotein U1 subunit 70 decreased influenza A replication and viral numbers.^77^ IRF‐3 decreased infection by HCV, West Nile virus, and Dengue virus.^76^, ^78^ In the red region, HSF1 decreased HCV replication.^76^ protein kinase CAMP‐activated catalytic subunit alpha decreased VSV infection.^79^ Signal transducer and activator of transcription 6 decreased IL‐8 expression. RAP2A, MINK1, and GNAS are regulatory proteins in the RAS pathway. RAP2A and MINK1 were detected only in the HP‐PRRSV group, whereas GNAS was detected only in the AP‐PRRSV group, in accordance with a previous report that stimulation of RAS increases the replication ability of HCV by reducing IFN‐JAK‐STAT pathway activity.^80^ We proposed that PRRSV was also related to the RAS pathway, and differed between HP‐PRRSV and AP‐PRRSV.

CREB‐binding protein (CREBBP) is composed of double‐stranded RNA‐activated transcription factor with IRF‐3, and double‐stranded RNA‐activated transcription factor is activated in many virus‐infected cells to promote apoptosis.^81^, ^82^ CREBBP may interact with human herpes virus 8 vIRF‐1, which could inhibit the binding of CREBBP to IRF‐3.^83^ Our results showed that IRF‐3 abundance was tenfold lower in the HP‐PRRSV than the AP‐PRRSV group, which we proposed was a protective mechanism of PRRSV to escape from host immunity and ensure survival after viral infection. The ability of HP‐PRRSV HuN4 to induce cell apoptosis is stronger than classical PRRSV (CH‐1a) in immune organs and lungs of piglets.^84^ We concluded that HP‐PRRSV might interact with CREBBP and decrease IRF‐3 expression to escape from host immunity and cause severe damage to the cell, highlighting a difference from AP‐PRRSV.

Notable differences exist during the infection of AP‐PRRSV and HP‐PRRSV. HP‐PRRSV could inhibit host immune function and evade the immune response via unknown mechanism.^85^, ^86^ Label‐free MS was performed using AP‐PRRSV‐infected and HP‐PRRSV‐infected PAMs. This is the first attempt to explore the differential characteristics between HP‐PRRSV and its attenuated PRRSV infected PAMs focusing on membrane proteins. By analyzing detected proteins in HP‐infected, AP‐PRRSV‐infected, and control group, proteins related to the immune response or virus replication were identified that may elucidate unique pathways used by different virulent PRRSVs for cell entry, virus replication, and immune escape mechanisms. Researches on these detected proteins will help with the elucidation of the identity, the expression abundance, and significance of them, future study will be focused on functions of these key membrane proteins to deepen our understanding of differential mechanisms between HP‐PRRSV and AP‐PRRSV infection.

**Abbreviations**
ADSL: adenylosuccinate lyase
AP: attenuated pathogenic
BP: biological process
CC: cellular component
CD: cluster of differentiation
CREBBP: CREB‐binding protein
CSFV: classical swine fever virus
GM‐CSF: granulocyte‐macrophage colony‐stimulating factor
GO: gene ontology
HCV: hepatitis C virus
HIV: human immunodeficiency virus
HP: highly pathogenic
LC‐MS/MS: liquid chromatography‐tandem MS
LFQPlabel‐free quantitative proteomicsMF: label‐free quantitative proteomicsMFmolecular function
MOI: multiplicity of infections
MP: membrane protein
MYH9: myosin 9
PAMs: pulmonary alveolar macrophages
PCV2: porcine circovirus 2
PPIB: peptidyl‐prolyl cis‐trans isomerase B
PRRSV: porcine reproductive and respiratory syndrome virus
TCID_50_: tissue culture infective dose
v‐ATPase: vacuolar ATPase
VCL: vinculin
VIM: vimentin
VSV: vesicular stomatitis virus

## Conflict of Interest

The authors declare that they have no conflicts of interest associated with this report.

## Supporting information

Supplementary material

## References

[1] J. J. Meulenberg , M. M. Hulst , E. J. de Meijer , P. L. Moonen , A. den Besten , E. P. de Kluyver , G. Wensvoort , R. J. Moormann , Virology 1993, 192, 62. 8517032 10.1006/viro.1993.1008 PMC7173055

[2] J. Lv , J. Zhang , Z. Sun , W. Liu , S. Yuan , J. Gen. Virol. 2008, 89, 2075. 18753215 10.1099/vir.0.2008/001529-0

[3] K. Tian , X. Yu , T. Zhao , Y. Feng , Z. Cao , C. Wang , Y. Hu , X. Chen , D. Hu , X. Tian , D. Liu , S. Zhang , X. Deng , Y. Ding , L. Yang , Y. Zhang , H. Xiao , M. Qiao , B. Wang , L. Hou , X. Wang , X. Yang , L. Kang , M. Sun , P. Jin , S. Wang , Y. Kitamura , J. Yan , G. F. Gao , PloS ONE 2007, 2, e526. 17565379 10.1371/journal.pone.0000526 PMC1885284

[4] J. J. Meulenberg , J. N. Bos‐de Ruijter , R. van de Graaf , G. Wensvoort , R. J. Moormann , J. Virol. 1998, 72, 380. 9420236 10.1128/jvi.72.1.380-387.1998 PMC109385

[5] A. O. Pasternak , W. J. Spaan , E. J. Snijder , J. Gen. Virol. 2006, 87, 1403. 16690906 10.1099/vir.0.81611-0

[6] H. S. Nielsen , G. Liu , J. Nielsen , M. B. Oleksiewicz , A. Botner , T. Storgaard , K. S. Faaberg , J. Virol. 2003, 77, 3702. 12610145 10.1128/JVI.77.6.3702-3711.2003 PMC149535

[7] Z. Sun , C. Liu , F. Tan , F. Gao , P. Liu , A. Qin , S. Yuan , Virus Res. 2010, 154, 38. 20833212 10.1016/j.virusres.2010.08.027 PMC7114379

[8] Z. Sun , J. Y. Wang , J. W. Zhang , A. J. Qin , S. S. Yuan , Acta Microbiol. Sin. 2007, 47, 774. 18062247

[9] K. KK , Am. Assoc. Swine Pract. Newsl. 1989, 1, 1.

[10] G. Z. Tong , Y. J. Zhou , X. F. Hao , Z. J. Tian , T. Q. An , H. J. Qiu , Emerg. Infect. Dis. 2007, 13, 1434. 18252136 10.3201/eid1309.070399 PMC2857295

[11] T. Q. An , Z. J. Tian , C. L. Leng , J. M. Peng , G. Z. Tong , Emerg. Infect. Dis. 2011, 17, 1782. 21888830 10.3201/eid1709.110411 PMC3322091

[12] X. Leng , Z. Li , M. Xia , X. Li , F. Wang , W. Wang , X. Zhang , H. Wu , Vet. Microbiol. 2012, 157, 50. 22245402 10.1016/j.vetmic.2011.12.012

[13] Y. J. Zhou , J. P. Zhu , T. Zhou , Q. Cheng , L. X. Yu , Y. X. Wang , S. Yang , Y. F. Jiang , W. Tong , F. Gao , H. Yu , G. X. Li , G. Z. Tong , PloS ONE 2014, 9, e85767. 24465692 10.1371/journal.pone.0085767 PMC3897507

[14] H. S. Kim , J. Kwang , I. J. Yoon , H. S. Joo , M. L. Frey , Arch. Virol. 1993, 133, 477. 8257302 10.1007/BF01313785

[15] P. G. Halbur , P. S. Paul , M. L. Frey , J. Landgraf , K. Eernisse , X. J. Meng , J. J. Andrews , M. A. Lum , J. A. Rathje , Vet. Pathol. 1996, 33, 159. 8801709 10.1177/030098589603300205

[16] X. Duan , H. J. Nauwynck , M. B. Pensaert , Arch. Virol. 1997, 142, 2483. 9672608 10.1007/s007050050256 PMC7086874

[17] X. Duan , H. J. Nauwynck , M. B. Pensaert , Vet. Microbiol. 1997, 56, 9. 9228678 10.1016/S0378-1135(96)01347-8

[18] C. L. Jolly , Q. J. Sattentau , Adv. Exp. Med. Biol. 2013, 790, 1. 23884583 10.1007/978-1-4614-7651-1_1

[19] H. J. Nauwynck , X. Duan , H. W. Favoreel , P. Van Oostveldt , M. B. Pensaert , J Gen Virol 1999, 80, 297. 10073688 10.1099/0022-1317-80-2-297

[20] J. Zheng , R. J. Sugrue , K. Tang , Anal. Chim. Acta. 2011, 702, 149. 21839192 10.1016/j.aca.2011.06.045 PMC7094357

[21] Y. Z. Xu , Y. J. Zhou , S. R. Zhang , W. Tong , L. Li , Y. F. Jiang , G. Z. Tong , Virol. J. 2012, 9, 141. 22856599 10.1186/1743-422X-9-141 PMC3422162

[22] Y. Z. Xu , Y. J. Zhou , S. R. Zhang , Y. F. Jiang , W. Tong , H. Yu , G. Z. Tong , Vet. Microbiol. 2012, 159, 1. 22513707 10.1016/j.vetmic.2012.03.015

[23] G. Wensvoort , C. Terpstra , J. M. Pol , E. A. ter Laak , M. Bloemraad , E. P. de Kluyver , C. Kragten , L. van Buiten , A. den Besten , F. Wagenaar , et al., Vet. Q. 1991, 13, 121. 1835211 10.1080/01652176.1991.9694296

[24] C. M. Ernst , P. Staubitz , N. N. Mishra , S. J. Yang , G. Hornig , H. Kalbacher , A. S. Bayer , D. Kraus , A. Peschel , PLoS Pathog. 2009, 5, e1000660. 19915718 10.1371/journal.ppat.1000660 PMC2774229

[25] E. Nakamura , K. Kozaki , H. Tsuda , E. Suzuki , A. Pimkhaokham , G. Yamamoto , T. Irie , T. Tachikawa , T. Amagasa , J. Inazawa , I. Imoto , Cancer Sci. 2008, 99, 1390. 18452558 10.1111/j.1349-7006.2008.00838.x PMC11158686

[26] K. J. Wiechelman , R. D. Braun , J. D. Fitzpatrick , Anal. Biochem. 1988, 175, 231. 3245570 10.1016/0003-2697(88)90383-1

[27] A. Michalski , E. Damoc , J. P. Hauschild , O. Lange , A. Wieghaus , A. Makarov , N. Nagaraj , J. Cox , M. Mann , S. Horning , Mol. Cell. Proteomics 2011, 10, M111 011015. 10.1074/mcp.M111.011015 PMC3284220 21642640

[28] Y. F. Jiang , T. Q. Xia , Y. J. Zhou , L. X. Yu , S. Yang , Q. F. Huang , L. W. Li , F. Gao , Z. H. Qu , W. Tong , G. Z. Tong , Vet. Microbiol. 2015, 179, 242. 26162970 10.1016/j.vetmic.2015.06.015

[29] Z. J. Tian , T. Q. An , Y. J. Zhou , J. M. Peng , S. P. Hu , T. C. Wei , Y. F. Jiang , Y. Xiao , G. Z. Tong , Vet. Microbiol. 2009, 138, 34. 19339125 10.1016/j.vetmic.2009.03.003

[30] N. Music , C. A. Gagnon , Anim. Health Res. Rev. 2010, 11, 135. 20388230 10.1017/S1466252310000034

[31] S. Zhang , Y. Zhou , Y. Jiang , G. Li , L. Yan , H. Yu , G. Tong , Virol. J. 2011, 8, 410. 21851649 10.1186/1743-422X-8-410 PMC3168427

[32] P. D. Simoncic , A. Lee‐Loy , D. L. Barber , M. L. Tremblay , C. J. McGlade , Curr. Biol. 2002, 12, 446. 11909529 10.1016/s0960-9822(02)00697-8

[33] J. ten Hoeve , M. de Jesus Ibarra‐Sanchez , Y. Fu , W. Zhu , M. Tremblay , M. David , K. Shuai , Mol. Cell. Biol. 2002, 22, 5662. 12138178 10.1128/MCB.22.16.5662-5668.2002 PMC133976

[34] F. Wiede , B. J. Shields , S. H. Chew , K. Kyparissoudis , C. van Vliet , S. Galic , M. L. Tremblay , S. M. Russell , D. I. Godfrey , T. Tiganis , J. Clin. Invest. 2011, 121, 4758. 22080863 10.1172/JCI59492 PMC3226006

[35] R. Brooks , N. Kizer , L. Nguyen , A. Jaishuen , K. Wanat , E. Nugent , P. Grigsby , J. E. Allsworth , J. S. Rader , Gynecol. Oncol. 2010, 116, 539. 19906411 10.1016/j.ygyno.2009.09.037 PMC2822070

[36] H. Kurachi , Y. Wada , N. Tsukamoto , M. Maeda , H. Kubota , M. Hattori , K. Iwai , N. Minato , J. Biol. Chem. 1997, 272, 28081. 9346962 10.1074/jbc.272.44.28081

[37] N. P. Gerard , C. Gerard , Nature 1991, 349, 614. 1847994 10.1038/349614a0

[38] Z. Chen , X. Zhang , N. C. Gonnella , T. C. Pellas , W. C. Boyar , F. Ni , J. Biol. Chem. 1998, 273, 10411. 9553099 10.1074/jbc.273.17.10411

[39] T. Christophe , M. J. Rabiet , M. Tardif , M. D. Milcent , F. Boulay , J. Biol. Chem. 2000, 275, 1656. 10636859 10.1074/jbc.275.3.1656

[40] J. A. DeMartino , G. Van Riper , S. J. Siciliano , C. J. Molineaux , Z. D. Konteatis , H. Rosen , M. S. Springer , J. Biol. Chem. 1994, 269, 14446. 8182049

[41] P. N. Monk , M. D. Barker , L. J. Partridge , J. E. Pease , J. Biol. Chem. 1995, 270, 16625. 7622471 10.1074/jbc.270.28.16625

[42] B. Postma , M. J. Poppelier , J. C. van Galen , E. R. Prossnitz , J. A. van Strijp , C. J. de Haas , K. P. van Kessel , J. Immunol. 2004, 172, 6994. 15153520 10.4049/jimmunol.172.11.6994

[43] S. Weitzer , J. Martinez , Nature 2007, 447, 222. 17495927 10.1038/nature05777

[44] A. Watanabe , M. Tatematsu , K. Saeki , S. Shibata , H. Shime , A. Yoshimura , C. Obuse , T. Seya , M. Matsumoto , J. Biol. Chem. 2011, 286, 10702. 21266579 10.1074/jbc.M110.185793 PMC3060521

[45] K. Saeki , Y. Miura , D. Aki , T. Kurosaki , A. Yoshimura , EMBO J 2003, 22, 3015. 12805216 10.1093/emboj/cdg293 PMC162145

[46] K. Shiba , N. Suzuki , K. Shigesada , Y. Namba , P. Schimmel , T. Noda , Proc. Natl. Acad. Sci. USA 1994, 91, 7435. 8052601 10.1073/pnas.91.16.7435 PMC44415

[47] R. C. Nichols , J. Blinder , S. I. Pai , Q. Ge , I. N. Targoff , P. H. Plotz , P. Liu , Genomics 1996, 36, 210. 8812440 10.1006/geno.1996.0449

[48] J. L. Bays , X. Peng , C. E. Tolbert , C. Guilluy , A. E. Angell , Y. Pan , R. Superfine , K. Burridge , K. A. DeMali , J. Cell. Biol. 2014, 205, 251. 24751539 10.1083/jcb.201309092 PMC4003237

[49] N. Machida , M. Umikawa , K. Takei , N. Sakima , B. E. Myagmar , K. Taira , H. Uezato , Y. Ogawa , K. Kariya , J. Biol. Chem. 2004, 279, 15711. 14966141 10.1074/jbc.C300542200

[50] H. Nonaka , K. Takei , M. Umikawa , M. Oshiro , K. Kuninaka , M. Bayarjargal , T. Asato , Y. Yamashiro , Y. Uechi , S. Endo , T. Suzuki , K. Kariya , Biochem. Biophys. Res. Commun. 2008, 377, 573. 18930710 10.1016/j.bbrc.2008.10.038

[51] M. Torti , A. Bertoni , I. Canobbio , F. Sinigaglia , E. G. Lapetina , C. Balduini , J. Cell. Biochem. 1999, 75, 675. 10572250 10.1002/(sici)1097-4644(19991215)75:4<675::aid-jcb13>3.0.co;2-m

[52] J. M. Enserink , A. E. Christensen , J. de Rooij , M. van Triest , F. Schwede , H. G. Genieser , S. O. Doskeland , J. L. Blank , J. L. Bos , Nat. Cell Biol. 2002, 4, 901. 12402047 10.1038/ncb874

[53] J. Menetrey , J. Cherfils , Proteins 1999, 37, 465. 10591105 10.1002/(sici)1097-0134(19991115)37:3<465::aid-prot13>3.0.co;2-o

[54] Y. Bi , X. K. Guo , H. Zhao , L. Gao , L. Wang , J. Tang , W. H. Feng , J. Virol. 2014, 88, 2810. 24352469 10.1128/JVI.03205-13 PMC3958105

[55] J. M. Weidner , D. Jiang , X. B. Pan , J. Chang , T. M. Block , J. T. Guo , J. Virol. 2010, 84, 12646. 20943977 10.1128/JVI.01328-10 PMC3004348

[56] I. C. Huang , C. C. Bailey , J. L. Weyer , S. R. Radoshitzky , M. M. Becker , J. J. Chiang , A. L. Brass , A. A. Ahmed , X. Chi , L. Dong , L. E. Longobardi , D. Boltz , J. H. Kuhn , S. J. Elledge , S. Bavari , M. R. Denison , H. Choe , M. Farzan , PLoS Pathog. 2011, 7, e1001258. 21253575 10.1371/journal.ppat.1001258 PMC3017121

[57] A. L. Brass , I. C. Huang , Y. Benita , S. P. John , M. N. Krishnan , E. M. Feeley , B. J. Ryan , J. L. Weyer , L. van der Weyden , E. Fikrig , D. J. Adams , R. J. Xavier , M. Farzan , S. J. Elledge , Cell 2009, 139, 1243. 20064371 10.1016/j.cell.2009.12.017 PMC2824905

[58] G. Jansen , P. Maattanen , A. Y. Denisov , L. Scarffe , B. Schade , H. Balghi , K. Dejgaard , L. Y. Chen , W. J. Muller , K. Gehring , D. Y. Thomas , Mol. Cell. Proteomics 2012, 11, 710. 22665516 10.1074/mcp.M111.016550 PMC3434782

[59] K. Watashi , N. Ishii , M. Hijikata , D. Inoue , T. Murata , Y. Miyanari , K. Shimotohno , Mol. Cell. 2005, 19, 111. 15989969 10.1016/j.molcel.2005.05.014

[60] J. G. Calvert , D. E. Slade , S. L. Shields , R. Jolie , R. M. Mannan , R. G. Ankenbauer , S. K. Welch , J. Virol. 2007, 81, 7371. 17494075 10.1128/JVI.00513-07 PMC1933360

[61] E. R. Jusa , Y. Inaba , M. Kouno , O. Hirose , Am. J. Vet. Res. 1997, 58, 488. 9140556

[62] K. Shanmukhappa , J. K. Kim , S. Kapil , Virol. J. 2007, 4, 62. 17572908 10.1186/1743-422X-4-62 PMC1906853

[63] P. L. Delputte , H. J. Nauwynck , J. Virol. 2004, 78, 8094. 15254181 10.1128/JVI.78.15.8094-8101.2004 PMC446125

[64] J. K. Kim , A. M. Fahad , K. Shanmukhappa , S. Kapil , J. Virol. 2006, 80, 689. 16378972 10.1128/JVI.80.2.689-696.2006 PMC1346842

[65] J. Gao , S. Xiao , Y. Xiao , X. Wang , C. Zhang , Q. Zhao , Y. Nan , B. Huang , H. Liu , N. Liu , J. Lv , T. Du , Y. Sun , Y. Mu , G. Wang , S. F. Syed , G. Zhang , J. A. Hiscox , I. Goodfellow , E. M. Zhou , Sci. Rep. 2016, 6, 25120. 27112594 10.1038/srep25120 PMC4845007

[66] E. E. Schmidt , O. Pelz , S. Buhlmann , G. Kerr , T. Horn , M. Boutros , update. Nucleic Acids Res. 2013, 41, D1021. 23193271 10.1093/nar/gks1170 PMC3531141

[67] N. Warner , A. Burberry , L. Franchi , Y. G. Kim , C. McDonald , M. A. Sartor , G. Nunez , Sci. Signal. 2013, 6, rs3. 23322906 10.1126/scisignal.2003305 PMC3887559

[68] Y. S. Ooi , K. M. Stiles , C. Y. Liu , G. M. Taylor , M. Kielian , PLoS Pathog. 2013, 9, e1003835. 24367265 10.1371/journal.ppat.1003835 PMC3868536

[69] I. Aydin , S. Weber , B. Snijder , P. Samperio Ventayol , A. Kuhbacher , M. Becker , P. M. Day , J. T. Schiller , M. Kann , L. Pelkmans , A. Helenius , M. Schelhaas , PLoS Pathog. 2014, 10, e1004162. 24874089 10.1371/journal.ppat.1004162 PMC4038628

[70] R. Konig , Y. Zhou , D. Elleder , T. L. Diamond , G. M. Bonamy , J. T. Irelan , C. Y. Chiang , B. P. Tu , P. D. De Jesus , C. E. Lilley , S. Seidel , A. M. Opaluch , J. S. Caldwell , M. D. Weitzman , K. L. Kuhen , S. Bandyopadhyay , T. Ideker , A. P. Orth , L. J. Miraglia , F. D. Bushman , J. A. Young , S. K. Chanda , Cell 2008, 135, 49. 18854154 10.1016/j.cell.2008.07.032 PMC2628946

[71] A. W. Tai , Y. Benita , L. F. Peng , S. S. Kim , N. Sakamoto , R. J. Xavier , R. T. Chung , Cell Host Microbe 2009, 5, 298. 19286138 10.1016/j.chom.2009.02.001 PMC2756022

[72] V. Betapudi , PloS ONE 2010, 5, e8560. 20052411 10.1371/journal.pone.0008560 PMC2797395

[73] C. Le Clainche , S. P. Dwivedi , D. Didry , M. F. Carlier , J. Biol. Chem. 2010, 285, 23420. 20484056 10.1074/jbc.M110.102830 PMC2906333

[74] C. Brown , S. G. Morham , D. Walsh , M. H. Naghavi , J. Mol. Biol. 2011, 410, 761. 21763488 10.1016/j.jmb.2011.03.076

[75] Y. Doyon , W. Selleck , W. S. Lane , S. Tan , J. Cote , Mol. Cell. Biol. 2004, 24, 1884. 14966270 10.1128/MCB.24.5.1884-1896.2004 PMC350560

[76] Q. Li , A. L. Brass , A. Ng , Z. Hu , R. J. Xavier , T. J. Liang , S. J. Elledge , Proc. Natl. Acad. Sci. USA 2009, 106, 16410. 19717417 10.1073/pnas.0907439106 PMC2752535

[77] A. Karlas , N. Machuy , Y. Shin , K. P. Pleissner , A. Artarini , D. Heuer , D. Becker , H. Khalil , L. A. Ogilvie , S. Hess , A. P. Maurer , E. Muller , T. Wolff , T. Rudel , T. F. Meyer , Nature 2010, 463, 818. 20081832 10.1038/nature08760

[78] M. N. Krishnan , A. Ng , B. Sukumaran , F. D. Gilfoy , P. D. Uchil , H. Sultana , A. L. Brass , R. Adametz , M. Tsui , F. Qian , R. R. Montgomery , S. Lev , P. W. Mason , R. A. Koski , S. J. Elledge , R. J. Xavier , H. Agaisse , E. Fikrig , Nature 2008, 455, 242. 18690214 10.1038/nature07207 PMC3136529

[79] L. Pelkmans , E. Fava , H. Grabner , M. Hannus , B. Habermann , E. Krausz , M. Zerial , Nature 2005, 436, 78. 15889048 10.1038/nature03571

[80] Q. Zhang , R. Gong , J. Qu , Y. Zhou , W. Liu , M. Chen , Y. Liu , Y. Zhu , J. Wu , J. Virol. 2012, 86, 1544. 22114332 10.1128/JVI.00688-11 PMC3264379

[81] B. K. Weaver , K. P. Kumar , N. C. Reich , Mol. Cell. Biol. 1998, 18, 1359. 9488451 10.1128/mcb.18.3.1359 PMC108849

[82] B. K. Weaver , O. Ando , K. P. Kumar , N. C. Reich , FASEB J 2001, 15, 501. 11156966 10.1096/fj.00-0222com

[83] R. Lin , P. Genin , Y. Mamane , M. Sgarbanti , A. Battistini , W. J. Harrington, Jr. , G. N. Barber , J. Hiscott , Oncogene 2001, 20, 800. 11314014 10.1038/sj.onc.1204163

[84] G. Wang , Y. He , Y. Tu , Y. Liu , E. M. Zhou , Z. Han , C. Jiang , S. Wang , W. Shi , X. Cai , Virol. J. 2014, 11, 2. 24393149 10.1186/1743-422X-11-2 PMC3892014

[85] D. Bao , R. Wang , S. Qiao , B. Wan , Y. Wang , M. Liu , X. Shi , J. Guo , G. Zhang , Vet. Immunol. Immunopathol. 2013, 156, 128. 24099951 10.1016/j.vetimm.2013.09.006

[86] Y. Sun , M. Han , C. Kim , J. G. Calvert , D. Yoo , Viruses 2012, 4, 424. 22590680 10.3390/v4040424 PMC3347317

[87] Y. Meng , F. Liu , C. Pang , S. Fan , M. Song , D. Wang , W. Li , S. Yu , J. Proteome Res. 2011, 10, 5416. 22029526 10.1021/pr200671d

[88] S. C. Johnson , Psychometrika 1967, 32, 241. 5234703 10.1007/BF02289588

