# Supplementary material for: Label‐Free Quantitative Proteomic Analysis of Differentially Expressed Membrane Proteins of Pulmonary Alveolar Macrophages Infected with Highly Pathogenic Porcine Reproductive and Respiratory Syndrome Virus and Its Attenuated Strain
Source: Proteomics. 2017 Nov 24;17(23-24):1700101. doi: 10.1002/pmic.201700101 (PMC6084361; doi:10.1002/pmic.201700101)
Supplement: Supplementary file 1 — Supplementary material [file PMIC-17-1700101-s001.docx]

**Supplementary Material**

| **Protein Accession(Con/AP-PRRSV)** | **Gene Name** | **T test (Con/AP-PRRSV)** | **AP-PRRSV/Con** | **Protein Accession(Con/HP-PRRSV)** | **Gene Name** | **T test (Con/HP-PRRSV)** | **HP-PRRSV/Con** |
| --- | --- | --- | --- | --- | --- | --- | --- |
| A5GFX7 | CTSZ CH242-247L10.3-001 | 2.604E-06 | 0.244 | A5GFX7 | CTSZ CH242-247L10.3-001 | 1.143E-05 | 0.291 |
| F1SV36 | LOC100522887 | 1.307E-05 | 12.527 | F2Z5J9 | RAB11B | 2.422E-06 | 0.379 |
| A5A766 | GPNMB | 1.440E-05 | 0.300 | F1SV36 | LOC100522887 | 7.626E-04 | 8.876 |
| I3LS26 | PSAP | 1.446E-05 | 0.274 | A5A766 | GPNMB | 1.200E-05 | 0.204 |
| F1SC70 | CTSA | 1.998E-05 | 0.423 | I3LS26 | PSAP | 1.480E-05 | 0.254 |
| F1S895 | LOC100624528 | 2.696E-05 | 0.191 | F1SC70 | CTSA | 1.186E-05 | 0.304 |
| Q52NJ2 | RAB1A | 2.930E-05 | 0.489 | F1S895 | LOC100624528 | 1.263E-04 | 0.250 |
| F1RUL2 | PFKFB3 | 3.118E-05 | 10.922 | Q52NJ2 | RAB1A | 7.173E-05 | 0.486 |
| F1S9V1 | PLD4 | 4.420E-05 | 0.261 | F1RUL2 | PFKFB3 | 6.159E-05 | 6.638 |
| F1SDJ6 | ITSN2 | 4.864E-05 | 3.944 | F1S9V1 | PLD4 | 4.344E-03 | 0.199 |
| I3LM93 | TNPO1 | 8.151E-05 | 3.240 | F1SDJ6 | ITSN2 | 5.395E-04 | 3.012 |
| F1SS93 | CTSS | 1.055E-04 | 0.374 | I3LM93 | TNPO1 | 1.795E-04 | 3.386 |
| I3LGC2 | RRBP1 | 1.072E-04 | 4.033 | F1SS93 | CTSS | 2.694E-04 | 0.312 |
| B1PEY3 | H2A.Z | 1.129E-04 | 0.258 | I3LGC2 | RRBP1 | 4.635E-04 | 3.142 |
| F1SGD1 |  | 1.177E-04 | 26.510 | B1PEY3 | H2A.Z | 1.049E-04 | 0.193 |
| F1RKC8 | SDS | 1.202E-04 | 10.477 | F1SGD1 |  | 1.115E-04 | 23.352 |
| R4L6V8 | SP100 | 1.210E-04 | 8.819 | F1RKC8 | SDS | 4.492E-03 | 6.684 |
| I3LKA3 | PTPN6 | 1.291E-04 | 2.271 | I3LPP4 |  | 6.972E-04 | 0.131 |
| I3LPP4 |  | 1.323E-04 | 0.009 | F1SIV5 | COMMD1 | 2.391E-03 | 2.878 |
| F1SIV5 | COMMD1 | 1.369E-04 | 2.771 | F1RZA6 | RNF213 | 3.028E-05 | 6.232 |
| F1RZA6 | RNF213 | 1.488E-04 | 6.435 | F1SV22 | MACF1 | 1.421E-04 | 4.956 |
| F1SV22 | MACF1 | 1.541E-04 | 4.995 | F1S715 | FUCA2 | 2.811E-04 | 0.305 |
| F1S715 | FUCA2 | 1.861E-04 | 0.257 | I3LMG2 | IFI44L | 2.698E-04 | 12.849 |
| I3LMG2 | IFI44L | 2.008E-04 | 9.428 | P00377 | DHFR | 3.344E-04 | 0.401 |
| P00377 | DHFR | 2.063E-04 | 0.372 | F1RQZ0 | GRN | 1.652E-04 | 0.316 |
| F1RQZ0 | GRN | 2.326E-04 | 0.388 | D0G6R9 | CNBP | 6.841E-04 | 6.537 |
| D0G6R9 | CNBP | 2.400E-04 | 8.177 | F1SBP9 | MOV10 | 7.393E-04 | 2.906 |
| F1SBP9 | MOV10 | 2.496E-04 | 3.386 | F1RS76 | LOC100737431 | 9.979E-03 | 2.246 |
| F1SPG1 | H1FX | 2.716E-04 | 0.202 | F1SPG1 | H1FX | 1.803E-04 | 0.162 |
| F1SML4 | SND1 | 2.718E-04 | 4.086 | F1SML4 | SND1 | 2.800E-04 | 3.236 |
| O02799 | STAT2 | 2.729E-04 | 7.304 | O02799 | STAT2 | 2.237E-05 | 8.765 |
| F1RHG1 | CLUH | 2.854E-04 | 4.973 | F1RHG1 | CLUH | 3.557E-03 | 5.766 |
| B2LUG8 |  | 2.864E-04 | 5.218 | B2LUG8 |  | 1.975E-04 | 4.510 |
| F1S3J9 | ICAM1 | 3.294E-04 | 2.725 | F1S3J9 | ICAM1 | 4.106E-04 | 2.959 |
| F1RQR4 | EHD1 | 3.414E-04 | 9.381 | F1S8L9 | HNRNPU | 6.852E-05 | 0.475 |
| B3SP85 | IFI30 GILT | 3.540E-04 | 0.448 | F1RQR4 | EHD1 | 4.961E-03 | 5.368 |
| Q4TTS4 |  | 3.716E-04 | 0.178 | B3SP85 | IFI30 GILT | 1.936E-04 | 0.362 |
| P62802 |  | 4.199E-04 | 0.244 | F1SQS2 | TMPO | 5.036E-04 | 0.474 |
| I3L5A6 | WIPF1 | 5.398E-04 | 7.997 | Q4TTS4 |  | 1.455E-04 | 0.120 |
| Q53DY5 | HIST1H1D | 5.635E-04 | 0.220 | P62802 |  | 3.378E-04 | 0.225 |
| I3LPH5 | G3BP2 | 5.685E-04 | 3.825 | I3L5A6 | WIPF1 | 5.692E-03 | 4.100 |
| Q4U1U4 |  | 5.752E-04 | 0.417 | Q53DY5 | HIST1H1D | 2.081E-04 | 0.187 |
| F1RMQ6 | GVIN1 | 5.821E-04 | 4.390 | I3LPH5 | G3BP2 | 2.279E-03 | 3.655 |
| P21753 | TMSB10 | 6.029E-04 | 0.348 | Q4U1U4 |  | 7.066E-05 | 0.338 |
| I3LAQ1 | HIST2H2BF | 6.363E-04 | 0.314 | F1RMQ6 | GVIN1 | 1.265E-04 | 4.891 |
| A7VK01 | Mx1 | 7.338E-04 | 32.070 | I3LGI9 | GLB1 | 3.180E-04 | 0.431 |
| I3LFV4 | YBX1 | 7.465E-04 | 3.780 | P21753 | TMSB10 | 1.393E-04 | 0.273 |
| I6YP72 | GSK3B | 7.492E-04 | 2.658 | I3LAQ1 | HIST2H2BF | 1.088E-04 | 0.227 |
| I3LEB6 |  | 7.522E-04 | 0.204 | A7VK01 | Mx1 | 6.465E-05 | 35.222 |
| Q3ZD69 | LMNA | 8.187E-04 | 0.440 | I3LFV4 | YBX1 | 1.072E-03 | 4.035 |
| A8CYB8 | RIG-I | 8.534E-04 | 6.391 | I3LEB6 |  | 7.958E-04 | 0.215 |
| Q29599 | OAS1 | 8.648E-04 | 3.332 | Q3ZD69 | LMNA | 9.127E-04 | 0.403 |
| I3LBY0 | ARCN1 | 8.949E-04 | 2.044 | A8CYB8 | RIG-I | 4.475E-04 | 5.512 |
| F1RU33 | BANF1 | 9.242E-04 | 0.322 | Q29599 | OAS1 | 3.902E-03 | 2.645 |
| Q764M5 | STAT1 | 9.253E-04 | 2.565 | F1RU33 | BANF1 | 1.485E-03 | 0.370 |
| F1S445 | SQSTM1 | 9.742E-04 | 4.116 | Q764M5 | STAT1 | 2.031E-03 | 2.068 |
| F1RQ06 | FKBP2 | 9.748E-04 | 0.420 | Q29099 | PTBP1 PTB | 2.813E-04 | 0.469 |
| F1RRZ3 |  | 1.004E-03 | 4.029 | F1S445 | SQSTM1 | 5.113E-03 | 4.771 |
| I3LT48 | RANGAP1 | 1.042E-03 | 5.945 | F1RQ06 | FKBP2 | 1.170E-04 | 0.273 |
| F1S0L8 | MTDH | 1.052E-03 | 8.405 | F1RRZ3 |  | 1.162E-03 | 3.541 |
| I3LVP5 | PPP1R12A | 1.060E-03 | 6.055 | F1S0L8 | MTDH | 4.648E-04 | 6.375 |
| F1RWW4 | PDLIM5 | 1.097E-03 | 3.977 | I3LVP5 | PPP1R12A | 2.942E-04 | 6.325 |
| F2Z5W6 | LAMTOR1 | 1.135E-03 | 0.421 | F1RWW4 | PDLIM5 | 1.595E-03 | 3.764 |
| F1SIW0 | STAB1 | 1.135E-03 | 0.062 | F2Z5W6 | LAMTOR1 | 7.123E-04 | 0.394 |
| C3S7K6 | S100A9 | 1.140E-03 | 2.762 | F1SIW0 | STAB1 | 3.470E-03 | 0.183 |
| F1STE8 | EIF5B | 1.187E-03 | 2.924 | A1E295 | CTSB | 4.238E-04 | 0.440 |
| F1RYY7 | PDDC1 | 1.203E-03 | 5.673 | F1STE8 | EIF5B | 5.329E-03 | 2.335 |
| F1SNM7 | AGFG1 | 1.221E-03 | 2.779 | F1RYY7 | PDDC1 | 1.186E-03 | 4.773 |
| I3L5P8 |  | 1.241E-03 | 2.709 | F1SNM7 | AGFG1 | 4.368E-03 | 2.104 |
| B6VAP9 | APEX1 | 1.247E-03 | 0.349 | B6VAP9 | APEX1 | 9.378E-04 | 0.387 |
| P81405 | PSAP | 1.261E-03 | 0.387 | P81405 | PSAP | 8.972E-04 | 0.339 |
| F1C4E1 | IFIT3 | 1.278E-03 | 57.922 | F1C4E1 | IFIT3 | 1.397E-05 | 54.818 |
| F1RP77 | MYCBP2 | 1.307E-03 | 0.108 | F1RP77 | MYCBP2 | 1.485E-03 | 0.155 |
| I3LB61 | SAMD9 | 1.323E-03 | 4.676 | I3LB61 | SAMD9 | 6.035E-03 | 3.661 |
| A7VK00 | MX2 | 1.349E-03 | 2.301 | A7VK00 | MX2 | 1.930E-03 | 2.178 |
| Q2VL90 | CD163 M130 | 1.360E-03 | 0.416 | Q2VL90 | CD163 M130 | 6.308E-04 | 0.360 |
| B1PK15 |  | 1.382E-03 | 0.383 | B1PK15 |  | 4.730E-04 | 0.264 |
| F2Z5L2 | HIST2H2AA4 | 1.431E-03 | 0.214 | F2Z5L2 | HIST2H2AA4 | 8.250E-04 | 0.237 |
| I3LUJ5 | CNN2 | 1.437E-03 | 3.063 | I3LUJ5 | CNN2 | 8.421E-04 | 2.687 |
| F1SB60 | SOD2 | 1.440E-03 | 3.417 | F1SMC1 | LOC100621457 | 2.241E-03 | 0.288 |
| F1SMC1 | LOC100621457 | 1.472E-03 | 0.244 | F1RI46 | FAM98C | 5.208E-04 | 4.454 |
| F1RI46 | FAM98C | 1.498E-03 | 3.875 | F1SGG1 | KRT18 | 9.931E-04 | 0.202 |
| F1SMW8 | SERPINB10 | 1.547E-03 | 0.406 | F1RM10 | TCERG1 | 7.546E-04 | 0.270 |
| F1SGG1 | KRT18 | 1.550E-03 | 0.287 | Q4FAT7 | GUSB | 3.537E-04 | 0.242 |
| F1RM10 | TCERG1 | 1.569E-03 | 0.229 | F1SEY1 | MAN2B1 | 1.471E-03 | 0.240 |
| Q4FAT7 | GUSB | 1.585E-03 | 0.375 | I3LJ14 | LOC102165030 | 6.334E-04 | 0.217 |
| F1SEY1 | MAN2B1 | 1.614E-03 | 0.301 | F1SD70 |  | 5.989E-04 | 0.159 |
| I3LJ14 | LOC102165030 | 1.632E-03 | 0.346 | Q865A4 | PKR | 7.198E-06 | 10.361 |
| F1SD70 |  | 1.646E-03 | 0.307 | I3L700 | NUTF2 | 1.528E-03 | 0.301 |
| F1RJL6 | CLIP2 | 1.710E-03 | 4.419 | I3LKY5 | NMI | 7.392E-03 | 2.869 |
| F1S1L0 | BTK | 1.745E-03 | 2.115 | F2Z518 | LAMTOR2 | 5.861E-03 | 0.484 |
| K7GQW2 | S100A8 | 1.803E-03 | 2.877 | I3LDE1 | LOC100521234 | 1.095E-03 | 3.869 |
| Q865A4 | PKR | 1.815E-03 | 7.966 | F1S4U9 | EML4 | 2.624E-03 | 2.598 |
| I3L700 | NUTF2 | 1.843E-03 | 0.282 | F2Z557 | PABPC1 | 2.134E-03 | 2.645 |
| I3LKY5 | NMI | 1.867E-03 | 3.809 | F1RTV6 | SRP72 | 1.452E-03 | 3.436 |
| F1SUD4 | AIFM2 | 1.885E-03 | 2.694 | F1RZ09 | DEF6 | 1.727E-03 | 0.244 |
| F2Z518 | LAMTOR2 | 1.901E-03 | 0.324 | F1RVD4 | SEC31A | 1.439E-03 | 4.442 |
| F1RHH8 | PRPF8 | 1.921E-03 | 0.352 | I3LNS9 | FUCA1 | 1.665E-03 | 0.201 |
| I3LDE1 | LOC100521234 | 1.963E-03 | 4.487 | F1RUX1 | CORO1B | 4.515E-03 | 0.457 |
| F1S4U9 | EML4 | 2.003E-03 | 2.787 | G9K122 | MRC1 | 3.160E-03 | 0.206 |
| F2Z557 | PABPC1 | 2.021E-03 | 2.518 | F1S2J4 | EDC4 | 1.493E-03 | 3.360 |
| F1SUD6 | FAM120A | 2.058E-03 | 2.405 | I3LCI4 | ZFR | 7.663E-04 | 0.153 |
| F1RTV6 | SRP72 | 2.185E-03 | 3.218 | B6DT14 | PRPF3 | 3.964E-04 | 0.289 |
| F1RZ09 | DEF6 | 2.226E-03 | 0.490 | D3K5N7 | ELAVL1 | 7.040E-04 | 0.478 |
| F1RVD4 | SEC31A | 2.245E-03 | 5.819 | F1RLP9 | UBQLN4 | 4.153E-04 | 0.320 |
| I3LNS9 | FUCA1 | 2.278E-03 | 0.323 | F1RQ90 | RBMX | 6.621E-04 | 0.397 |
| F1RUX1 | CORO1B | 2.281E-03 | 0.362 | F1RW08 | SRP68 | 3.182E-03 | 3.086 |
| G9K122 | MRC1 | 2.310E-03 | 0.141 | F1SS29 | LOC100620327 SRP14 | 2.817E-03 | 2.948 |
| F1S2J4 | EDC4 | 2.376E-03 | 3.042 | Q95274 | TMSB4 | 2.863E-03 | 0.414 |
| I3LCI4 | ZFR | 2.401E-03 | 0.315 | F1SMZ0 | SP140 | 1.058E-03 | 3.836 |
| B6DT14 | PRPF3 | 2.426E-03 | 0.270 | I3LKM9 |  | 4.325E-03 | 0.339 |
| Q9N1F5 | GSTO1 | 2.615E-03 | 0.389 | F2Z5V3 | SNU13 | 3.320E-03 | 0.420 |
| F1S710 | CACYBP | 2.646E-03 | 2.409 | F1RFW9 | GATAD2B | 1.350E-03 | 0.207 |
| F1SBU9 | SAFB2 | 2.654E-03 | 0.240 | I3L6N8 |  | 2.233E-03 | 2.257 |
| I3L918 | LOC102157663 | 2.664E-03 | 0.415 | D4P3C2 | zap ZC3HAV1 | 4.874E-04 | 2.529 |
| K9IWA9 | DMXL2 | 2.669E-03 | 2.377 | P00795 | CTSD | 4.041E-03 | 0.350 |
| F1RLG4 | SRP19 | 2.904E-03 | 2.967 | A2SW51 | CD14 | 5.416E-04 | 3.276 |
| F1SKD5 | USP15 | 2.972E-03 | 4.319 | F1S619 | RBM15 | 1.995E-03 | 0.415 |
| F1RLP9 | UBQLN4 | 3.076E-03 | 0.463 | F1RGP1 | MYBBP1A | 1.179E-03 | 0.240 |
| K7GN17 | NCF4 | 3.166E-03 | 3.352 | F1SP18 | TARS | 4.865E-03 | 0.314 |
| F1RW08 | SRP68 | 3.189E-03 | 2.979 | F1SR85 | ABCB6 | 2.617E-05 | 0.116 |
| F1SS29 | LOC100620327 SRP14 | 3.264E-03 | 3.633 | Q52NJ3 | SAR1A | 5.482E-03 | 2.391 |
| I3LNC4 | STRAP | 3.296E-03 | 2.043 | K7GP99 | ILF3 | 9.715E-04 | 0.345 |
| I3LBD6 | OSBPL11 | 3.360E-03 | 2.629 | K9IVL7 | LRP1 | 3.578E-04 | 0.159 |
| Q95274 | TMSB4 | 3.421E-03 | 0.452 | P80272 | HMGN2 HMG17 | 3.869E-03 | 0.238 |
| F1SMZ0 | SP140 | 3.424E-03 | 4.106 | F1RMG0 | FAM111A | 4.893E-04 | 0.250 |
| I3LKM9 |  | 3.427E-03 | 0.322 | I3L5T3 | FBL | 4.648E-04 | 0.138 |
| F2Z5V3 | SNU13 | 3.454E-03 | 0.431 | Q866U4 |  | 8.880E-03 | 0.340 |
| F1RFW9 | GATAD2B | 3.482E-03 | 0.387 | I3LG67 | MARCKS | 2.416E-03 | 8.880 |
| K9J4M9 | COLEC12 | 3.525E-03 | 0.123 | I3LR17 | CORO1A | 1.186E-04 | 0.486 |
| D9MNC9 | CD43 | 3.569E-03 | 0.287 | F1SAR6 | SYNE3 | 3.008E-03 | 0.231 |
| I3L6N8 |  | 3.624E-03 | 2.089 | D0G6X8 | HEXB | 1.949E-03 | 0.377 |
| D4P3C2 | zap ZC3HAV1 | 3.694E-03 | 2.438 | I3LRW6 |  | 5.185E-03 | 2.245 |
| F1S4H0 | RBM47 | 3.774E-03 | 2.294 | K9J4W6 | ZNFX1 | 9.255E-04 | 41.473 |
| F1RJL2 | GTF2I | 3.941E-03 | 0.125 | F1S731 | STX11 | 2.777E-03 | 5.231 |
| P00795 | CTSD | 4.008E-03 | 0.393 | K7GSM4 | ARFGEF1 | 7.060E-03 | 3.251 |
| F1SIP0 | SEPT2 | 4.039E-03 | 2.138 | F1RKM0 | LMNB1 | 4.198E-03 | 0.126 |
| A2SW51 | CD14 | 4.088E-03 | 2.582 | F1S5M9 | ADD3 | 8.233E-03 | 3.545 |
| I3L6Q5 | HIST1H1B | 4.205E-03 | 0.103 | F1SBA6 | SS18 | 8.285E-03 | 0.285 |
| F1RGP1 | MYBBP1A | 4.224E-03 | 0.426 | F1SD60 | RFX1 | 5.802E-03 | 0.402 |
| F1SIE7 | EDC3 | 4.374E-03 | 2.842 | F1SQR1 | SYAP1 | 9.013E-04 | 2.155 |
| F1SP18 | TARS | 4.517E-03 | 0.301 | F1RKW7 | ZNF207 | 3.337E-03 | 0.416 |
| F1SJN5 | EIF2A | 4.646E-03 | 0.422 | K7GNY9 | VBP1 | 3.614E-04 | 2.590 |
| I3LF36 | SAP18 | 4.698E-03 | 0.233 | F1SMZ9 | SF3B1 | 4.789E-03 | 0.317 |
| A5JUQ8 | CRYZL1 | 4.859E-03 | 2.774 | F1S8R5 | SRP9 | 2.599E-03 | 3.507 |
| Q52NJ3 | SAR1A | 4.946E-03 | 2.518 | F1RQB6 | G3BP1 | 3.867E-04 | 5.409 |
| K9IVL7 | LRP1 | 5.174E-03 | 0.373 | F2Z576 | HIST1H3E | 6.303E-03 | 0.182 |
| P80272 | HMGN2 HMG17 | 5.338E-03 | 0.291 | I3LSU1 | NONO | 5.305E-03 | 0.347 |
| F1RMG0 | FAM111A | 5.421E-03 | 0.358 | F1RK53 | LOC100513346 | 5.551E-05 | 0.456 |
| F1S6V4 | EIF4G2 | 5.582E-03 | 3.439 | F1S7T1 | PRRC2C | 1.461E-03 | 4.928 |
| F1RKM1 | ALDH7A1 | 5.832E-03 | 0.360 | I3LG34 | RB1CC1 | 9.384E-03 | 4.473 |
| Q2XVP5 | MAPRE1 | 5.851E-03 | 2.232 | F1RKJ4 | YIF1B | 4.968E-03 | 0.451 |
| F1SV46 | MAP7D1 | 5.855E-03 | 2.237 | F1S7J6 | SAFB | 2.847E-03 | 0.435 |
| F1RU38 | SF3B2 | 6.197E-03 | 0.195 | I3LNE5 | SNRNP70 | 1.449E-03 | 0.276 |
| I3L5T3 | FBL | 6.206E-03 | 0.389 | F2Z4Z2 | GTF2A2 | 2.637E-03 | 0.326 |
| F1SML0 | GCC1 | 6.442E-03 | 2.496 | G3FP13 | PQBP1 | 3.473E-03 | 0.411 |
| Q866U4 |  | 6.443E-03 | 0.282 | F1RPK0 | LOC100154508 | 3.451E-03 | 0.133 |
| I3LG67 | MARCKS | 6.524E-03 | 14.788 | F1RUH5 | DEK | 8.782E-03 | 0.208 |
| Q8WNQ7 | GALNS | 6.577E-03 | 0.437 | F2Z560 | RAB5C | 6.170E-04 | 0.454 |
| F1SAR6 | SYNE3 | 6.690E-03 | 0.327 | F1RN44 | LAMP1 | 7.247E-03 | 0.394 |
| F1RMY1 | LRCH4 | 6.732E-03 | 0.346 | F1RZ82 | GAA | 8.325E-03 | 0.400 |
| F1SL19 | NFKBIZ | 6.733E-03 | 0.158 | F1RKC7 | PLBD2 | 1.685E-03 | 0.317 |
| D0G6X8 | HEXB | 6.845E-03 | 0.446 | K9IVK2 | RANBP2 | 4.649E-03 | 4.131 |
| F1SHM3 | SYNJ1 | 6.906E-03 | 9.531 | F1SQA9 | SEC13 | 3.659E-04 | 2.438 |
| I3LRW6 |  | 7.014E-03 | 2.131 | F1RSC3 | SCPEP1 | 9.891E-03 | 0.438 |
| F1S5Z3 | SARS | 7.024E-03 | 0.404 | F1SMX7 | SERPINB8 | 8.329E-03 | 0.465 |
| K9J4W6 | ZNFX1 | 7.117E-03 | 42.052 | F1SG44 | CHST11 | 9.728E-03 | 4.035 |
| F1S731 | STX11 | 7.190E-03 | 5.860 | I3LH62 | FXR1 | 8.860E-03 | 2.539 |
| K7GSM4 | ARFGEF1 | 7.200E-03 | 4.609 | K7GNR1 | IFIT2 | 1.364E-04 | 15.340 |
| B8XX91 | DAI | 7.365E-03 | 6.375 | K7GS54 | SMS | 4.713E-03 | 0.404 |
| I3LQN1 | MRAS | 7.385E-03 | 2.106 | F1SES5 | ASAH1 | 3.257E-03 | 0.354 |
| F1S9W6 | EPS15L1 | 7.648E-03 | 2.337 | I3LDV0 | DDX3Y | 3.785E-03 | 2.787 |
| F1SLA0 | ATP5B | 7.748E-03 | 2.195 | C4MXZ1 | ARF1 | 3.613E-03 | 2.209 |
| F2Z5V2 | SNRPG | 7.840E-03 | 0.271 | P0C8B5 |  | 3.157E-03 | 0.444 |
| F1RKM0 | LMNB1 | 7.866E-03 | 0.287 | I3L9G5 | BORCS7 | 1.109E-03 | 0.268 |
| F1SCM0 | NOP10 | 7.963E-03 | 0.316 | B2CCY7 | PRPF19 nmp-200 | 4.756E-03 | 0.345 |
| F1S5M9 | ADD3 | 7.979E-03 | 3.484 | F1RLQ2 | LMNA | 6.142E-03 | 0.385 |
| F1SBA6 | SS18 | 8.291E-03 | 0.382 | F1SIT7 | RPLP1 | 4.075E-03 | 2.001 |
| F1RIR2 | STX4 | 8.293E-03 | 0.208 | F1RZK8 | HDAC2 | 4.550E-03 | 0.291 |
| F1S593 | DNM2 | 8.487E-03 | 2.158 | K9IWG6 | SPTBN1 | 9.727E-03 | 0.173 |
| F1STG5 | MAP4K4 | 8.500E-03 | 3.057 | Q29073 | PTGR1 LTB4DH | 1.871E-03 | 0.322 |
| F1SD60 | RFX1 | 8.830E-03 | 0.489 | K9J6I8 | DOCK2 | 7.604E-03 | 2.109 |
| F1SBE8 | ARFGEF2 | 8.938E-03 | 2.421 | D0G7F1 | SCP2 | 8.367E-03 | 0.243 |
| F1SQR1 | SYAP1 | 9.125E-03 | 2.519 | F1S2G5 | ADK | 4.235E-03 | 0.345 |
| F1RRI2 | FRMD8 | 9.132E-03 | 0.392 | I3LMT3 | RBM14 | 3.029E-03 | 0.335 |
| Q6J1I8 | RNF114 ZNF313 | 9.186E-03 | 2.737 | F1S1D6 | ATP6V0A1 | 8.414E-03 | 0.308 |
| F1SMZ9 | SF3B1 | 9.310E-03 | 0.367 | Q9XSZ6 | FCER1G | 2.666E-04 | 0.342 |
| F2Z5B8 | LAMTOR3 | 9.394E-03 | 0.252 | F1S8G5 | THOP1 | 8.248E-03 | 0.313 |
| F1S8R5 | SRP9 | 9.482E-03 | 4.787 | F1RM65 | U2AF1L4 | 6.374E-03 | 0.266 |
| F1RQB6 | G3BP1 | 9.631E-03 | 3.633 | I3LLB7 |  | 6.584E-03 | 2.441 |
| F1SA77 | CHD2 | 9.865E-03 | 0.222 | I3LG73 | RSL1D1 | 4.532E-03 | 0.303 |
| F2Z576 | HIST1H3E | 9.871E-03 | 0.278 | F1S0B4 |  | 8.976E-03 | 0.426 |
| I3LSU1 | NONO | 9.904E-03 | 0.468 | K9J6M4 | UBR4 | 8.809E-04 | 0.231 |
| F1RK53 | LOC100513346 | 9.920E-03 | 0.441 | H9TUB5 |  | 8.317E-03 | 0.325 |
| F1S7T1 | PRRC2C | 9.939E-03 | 4.284 | F2Z5W4 |  | 9.345E-03 | 0.409 |
| I3LM39 | MB21D1 | N/A | vA+ Con- | F1RRR1 | GOSR2 | 6.562E-03 | 0.336 |
| P07802 | PRKAR1A | N/A | vA+ Con- | F1SA50 | HNRNPM | 9.347E-03 | 0.385 |
| P22952 |  | N/A | vA+ Con- | P12067 |  | 1.251E-04 | 0.367 |
| P26889 | IL1B | N/A | vA+ Con- | I3L9R9 | ATXN2L | 8.608E-04 | 8.475 |
| P26894 | CXCL8 IL8 | N/A | vA+ Con- | I3LHZ6 | DHX9 | 3.909E-03 | 0.466 |
| P36887 | PRKACA | N/A | vA+ Con- | I3LL97 | CSRP1 | 2.048E-03 | 0.476 |
| Q719N1 | SPAST SPG4 | N/A | vA+ Con- | I3LRD5 | EIF4H | 7.533E-03 | 3.361 |
| Q9MZU4 | RSAD2 CIG6 IRG6 | N/A | vA+ Con- | F1S4P6 | EIF3A EIF3S10 | 2.650E-04 | 2.012 |
| A2T1K6 | CD72 | N/A | vA+ Con- | F1RTW7 |  | 3.319E-03 | 2.513 |
| A7LCX1 | MDA5 | N/A | vA+ Con- | F1SGS6 | CAPRIN1 | 3.654E-04 | 7.260 |
| A9QT41 | IKBKG | N/A | vA+ Con- | F1SKT8 | PPWD1 | 5.415E-03 | 0.335 |
| A9QW81 | IFITM1 | N/A | vA+ Con- | I3LHF2 |  | 1.957E-03 | 5.723 |
| B2XWS1 | GBP1 | N/A | vA+ Con- | I3LU34 | DPP7 | 5.363E-03 | 0.130 |
| D3K5K4 | ACSL5 | N/A | vA+ Con- | F1S596 | PRKCSH | 1.242E-03 | 0.471 |
| E7EAX3 | IFITM3 | N/A | vA+ Con- | M3TYJ4 | MCM6 | 6.668E-03 | 0.333 |
| I3LTJ6 | TRRAP | N/A | vA+ Con- | F1S413 | MCMBP | 7.140E-03 | 0.365 |
| F1RG61 | TBC1D10B | N/A | vA+ Con- | F1ST28 | CD68 | 4.160E-03 | 0.351 |
| F1RHF0 | UCHL3 | N/A | vA+ Con- | Q5I240 | CREB1 | 2.476E-03 | 0.328 |
| F1RJ89 | RGCC | N/A | vA+ Con- | F1S129 | SEC24B | 2.554E-03 | 6.535 |
| F1RKK5 | PRRC1 | N/A | vA+ Con- | F1SUZ2 | NUP98 | 7.600E-04 | 0.180 |
| F1RRD4 | LOC100513756 | N/A | vA+ Con- | F1RN28 | PSPC1 | 9.532E-03 | 0.301 |
| F1RUL7 |  | N/A | vA+ Con- | Q5PYH3 | SAR1B | 2.507E-03 | 4.197 |
| F1RVF1 |  | N/A | vA+ Con- | A7Y521 | COPS4 | 2.396E-04 | 0.464 |
| F1S414 | INPP5F | N/A | vA+ Con- | F2Z513 | CFAP20 | 1.411E-03 | 0.433 |
| F1S4I1 | GOLM1 | N/A | vA+ Con- | F1RT87 | RAB2A | 3.642E-03 | 0.462 |
| F1S4P1 | HEATR5B | N/A | vA+ Con- | F1S3S7 | COMMD8 | 4.028E-03 | 2.038 |
| F1S567 | VCAM1 | N/A | vA+ Con- | Q6QRN9 | SLC25A6 ANT3 | 3.359E-04 | 0.235 |
| F1S6M0 | TRAPPC2L | N/A | vA+ Con- | P62197 | PSMC5 | 4.932E-03 | 2.642 |
| F1S9T2 | IFI44 | N/A | vA+ Con- | F1SF38 | MCM9 | 2.369E-05 | 0.059 |
| F1SAS2 | ACSS1 | N/A | vA+ Con- | F1RGM8 | ADAR | 8.982E-03 | 4.725 |
| J7FIC7 | IFIT1 | N/A | vA+ Con- | F2Z5F3 | BUB3 | 8.144E-03 | 0.483 |
| F1SDN2 | HADHB | N/A | vA+ Con- | F1S0A2 |  | 2.824E-03 | 0.280 |
| F1SE55 | C9orf72 | N/A | vA+ Con- | M3V851 | TFCP2 | 8.593E-04 | 0.324 |
| F1SEA2 | SLAIN2 | N/A | vA+ Con- | F2Z5V1 | TMA7 | 6.423E-03 | 0.204 |
| F1SES6 | LOC100514927 | N/A | vA+ Con- | F1SDJ8 | GLRX3 | 1.518E-04 | 6.291 |
| F1SEW7 | LOC100155842 C4H1orf52 | N/A | vA+ Con- | I3L7C1 | BORCS8 | 9.759E-03 | 0.384 |
| F1SGH5 | PDHB | N/A | vA+ Con- | A5D9P0 | RING1 SBAB-279G2.4-001 | 8.300E-04 | 0.342 |
| F1SH87 | LARP4 | N/A | vA+ Con- | F1SFX1 | UEVLD | 8.237E-03 | 0.452 |
| F1SJE2 | TJP2 | N/A | vA+ Con- | I3LDW0 | LOC100623007 | 6.379E-03 | 2.293 |
| F1SPP8 | CKAP4 | N/A | vA+ Con- | I3LHT9 | STX12 | 7.963E-03 | 0.497 |
| F1SRP3 | PARP12 | N/A | vA+ Con- | I3LJ55 | PARP3 | 1.696E-04 | 4.598 |
| F1SV88 | LOC100628001 | N/A | vA+ Con- | K7GS58 | CSTF2 | 9.179E-03 | 0.489 |
| F2Z565 | SLC25A5 | N/A | vA+ Con- | I3LSA6 | FCHO2 | 5.654E-03 | 0.257 |
| F4ZS20 | UBE2L6 | N/A | vA+ Con- | F1RRX4 | LCP2 | 7.510E-03 | 2.832 |
| I3L7U1 | BSDC1 | N/A | vA+ Con- | I3LP11 | KHDRBS1 | 4.365E-03 | 0.403 |
| I3LB04 | ISG20 | N/A | vA+ Con- | F1S4J1 | ZCCHC6 | 5.624E-03 | 2.467 |
| I3LR66 |  | N/A | vA+ Con- | F1S7L8 | SH3GL1 | 3.139E-03 | 0.441 |
| I3LRZ6 | LOC100737732 | N/A | vA+ Con- | F1SMN1 | CALU | 4.095E-03 | 0.344 |
| I3LSI3 | AK2 | N/A | vA+ Con- | F1RZV1 | BAG2 | 7.726E-03 | 5.160 |
| I3LSP2 | CCDC9 | N/A | vA+ Con- | F1REW9 | DENR | 4.868E-03 | 2.158 |
| I3LVP6 | ANKLE2 | N/A | vA+ Con- | F1S9U2 |  | 1.181E-03 | 5.889 |
| M3TYV9 | TCF20 | N/A | vA+ Con- | O77666 | TRIM26 ZNF173 | 2.536E-03 | 0.358 |
| M3VH87 | GAB2 | N/A | vA+ Con- | B8Y648 | UBE2L3 | 3.201E-03 | 2.142 |
| Q53DY6 |  | N/A | vA+ Con- | F1RGA7 | SART3 | 1.231E-04 | 0.356 |
| F2Z587 | HIST1H2AB HIST1H2AE | N/A | vA- Con+ | M3VH83 | DPP9 | 3.003E-07 | 45.199 |
| Q9TU45 | TYROBP DAP12 | N/A | vA- Con+ | F1S9X7 | FAM32A | 9.460E-03 | 0.491 |
| F1RWH7 | PNPO | N/A | vA- Con+ | P61958 | SUMO2 SMT3A SMT3H2 | 6.200E-03 | 0.453 |
| D2JUK1 | MITF | N/A | vA- Con+ | F1RGL6 | SCIMP | 8.306E-03 | 0.481 |
| Q29076 | POU2F1 OTF1 | N/A | vA- Con+ | F1SML9 | RIC1 | 1.107E-03 | 0.136 |
| I3LDD9 | BLOC1S4 | N/A | vA- Con+ | I3L5T1 | SGPP1 | 2.355E-03 | 0.323 |
| F1SR10 | CMAS | N/A | vA- Con+ | I3LD01 | FIS1 | 9.150E-05 | 0.380 |
| F1RUL1 | RBM17 | N/A | vA- Con+ | P26889 | IL1B | N/A | vH+ Con- |
| Q9GLE3 | CTSK | N/A | vA- Con+ | A9QW81 | IFITM1 | N/A | vH+ Con- |
| Q06AU2 | RAP2A | N/A | vA- Con+ | E7EAX3 | IFITM3 | N/A | vH+ Con- |
| F1RL77 | CSF1R | N/A | vA- Con+ | P22952 |  | N/A | vH+ Con- |
| F1S6R5 | WDR18 | N/A | vA- Con+ | F1SV88 | LOC100628001 | N/A | vH+ Con- |
| F1SV59 | ADPRHL2 | N/A | vA- Con+ | F4ZS15 | IFITM2 | N/A | vH+ Con- |
| M3UZ83 | PSMG2 | N/A | vA- Con+ | I3LAW4 |  | N/A | vH+ Con- |
| F1RTK6 | IGBP1 | N/A | vA- Con+ | J7FIC7 | IFIT1 | N/A | vH+ Con- |
| F1S912 | FKBP8 | N/A | vA- Con+ | P26894 | CXCL8 IL8 | N/A | vH+ Con- |
| F1S6C1 | THYN1 | N/A | vA- Con+ | F1SHI8 | FAM177A1 | N/A | vH+ Con- |
| F1SRI1 | RAB9A | N/A | vA- Con+ | F1RKK5 | PRRC1 | N/A | vH+ Con- |
| I3L9Y2 |  | N/A | vA- Con+ | Q53DY6 |  | N/A | vH+ Con- |
| Q69DL0 | C1QA | N/A | vA- Con+ | F4ZS20 | UBE2L6 | N/A | vH+ Con- |
| F1SH84 |  | N/A | vA- Con+ | P36887 | PRKACA | N/A | vH+ Con- |
| I3LAN0 | ZEB2 | N/A | vA- Con+ | I3L7U1 | BSDC1 | N/A | vH+ Con- |
| F1S2Z5 |  | N/A | vA- Con+ | I3L717 |  | N/A | vH+ Con- |
| F1SU88 |  | N/A | vA- Con+ | F1S9T2 | IFI44 | N/A | vH+ Con- |
| F1RQY4 | UBTF | N/A | vA- Con+ | F1RJ89 | RGCC | N/A | vH+ Con- |
| F1SCH5 | XAB2 | N/A | vA- Con+ | I3L8R1 | CLIC4 | N/A | vH+ Con- |
| C9K505 | CDK7 | N/A | vA- Con+ | F1SUH6 |  | N/A | vH+ Con- |
| I3L9C9 | SON | N/A | vA- Con+ | F1SSC4 | MPLKIP | N/A | vH+ Con- |
| I3LES5 | DUS2 | N/A | vA- Con+ | F1SH87 | LARP4 | N/A | vH+ Con- |
| K9J6J8 | NOTCH2 | N/A | vA- Con+ | F2Z5K9 | HIST2H3D LOC100622412 | N/A | vH+ Con- |
| I3L7B7 | DDX18 | N/A | vA- Con+ | Q9MZU4 | RSAD2 CIG6 IRG6 | N/A | vH+ Con- |
| F1SAB0 |  | N/A | vA- Con+ | F1S0Z5 | DNAJB14 | N/A | vH+ Con- |
| F1RKB5 | LOC100512219 | N/A | vA- Con+ | F1RR73 | NUDCD2 | N/A | vH+ Con- |
| F1S285 | COL14A1 | N/A | vA- Con+ | F1RRN1 | KLC4 | N/A | vH+ Con- |
| E1U8C5 | STAT6 | N/A | vA- Con+ | F1S6M0 | TRAPPC2L | N/A | vH+ Con- |
| F1SFG0 | NIN | N/A | vA- Con+ | F1SRP3 | PARP12 | N/A | vH+ Con- |
|  |  |  |  | F1RHF0 | UCHL3 | N/A | vH+ Con- |
|  |  |  |  | I3LLS0 |  | N/A | vH+ Con- |
|  |  |  |  | Q6IM70 | RTN4 | N/A | vH+ Con- |
|  |  |  |  | P07802 | PRKAR1A | N/A | vH+ Con- |
|  |  |  |  | F1STE3 | MITD1 | N/A | vH+ Con- |
|  |  |  |  | F1RWZ9 | FUNDC1 | N/A | vH+ Con- |
|  |  |  |  | A0A077ETG0 | TRIM56 | N/A | vH+ Con- |
|  |  |  |  | I3LP62 | RPL22L1 | N/A | vH+ Con- |
|  |  |  |  | I3LNC9 | LOC100739502 | N/A | vH+ Con- |
|  |  |  |  | F1S567 | VCAM1 | N/A | vH+ Con- |
|  |  |  |  | P33198 | IDH2 | N/A | vH+ Con- |
|  |  |  |  | I3LSI3 | AK2 | N/A | vH+ Con- |
|  |  |  |  | I3LMK9 |  | N/A | vH+ Con- |
|  |  |  |  | F1RLM7 | KIAA0907 | N/A | vH+ Con- |
|  |  |  |  | F1RGK5 | TPM3 | N/A | vH+ Con- |
|  |  |  |  | F1RTS8 | OGFRL1 | N/A | vH+ Con- |
|  |  |  |  | F1RNX2 | PDCD5 | N/A | vH+ Con- |
|  |  |  |  | I3LR32 | CCT5 | N/A | vH+ Con- |
|  |  |  |  | F1SCU6 | PUM2 | N/A | vH+ Con- |
|  |  |  |  | F1SE55 | C9orf72 | N/A | vH+ Con- |
|  |  |  |  | F1S1F6 | BECN1 | N/A | vH+ Con- |
|  |  |  |  | I3LSP2 | CCDC9 | N/A | vH+ Con- |
|  |  |  |  | F2Z5Q7 | NAA50 | N/A | vH+ Con- |
|  |  |  |  | F1SGJ0 | PXK | N/A | vH+ Con- |
|  |  |  |  | A7LCX1 | MDA5 | N/A | vH+ Con- |
|  |  |  |  | F2Z565 | SLC25A5 | N/A | vH+ Con- |
|  |  |  |  | F1SEA2 | SLAIN2 | N/A | vH+ Con- |
|  |  |  |  | I3LFS7 | PIP4K2B | N/A | vH+ Con- |
|  |  |  |  | F1SSG2 | TBC1D2 | N/A | vH+ Con- |
|  |  |  |  | F1RPN9 | ME2 | N/A | vH+ Con- |
|  |  |  |  | J7FJH8 | IFIT2 | N/A | vH+ Con- |
|  |  |  |  | I3LVD6 | UMPS | N/A | vH+ Con- |
|  |  |  |  | A2T1K6 | CD72 | N/A | vH+ Con- |
|  |  |  |  | F1SJE2 | TJP2 | N/A | vH+ Con- |
|  |  |  |  | B2XWS1 | GBP1 | N/A | vH+ Con- |
|  |  |  |  | D3K5K4 | ACSL5 | N/A | vH+ Con- |
|  |  |  |  | F1RPY5 | MARK2 | N/A | vH+ Con- |
|  |  |  |  | F1SPP8 | CKAP4 | N/A | vH+ Con- |
|  |  |  |  | F1RFI2 | AP1B1 | N/A | vH+ Con- |
|  |  |  |  | F1RRD4 | LOC100513756 | N/A | vH+ Con- |
|  |  |  |  | F1SBN7 | ATP5F1 | N/A | vH+ Con- |
|  |  |  |  | I3LR66 |  | N/A | vH+ Con- |
|  |  |  |  | I3LEQ6 | LOC102166414 | N/A | vH+ Con- |
|  |  |  |  | F1SUF6 | IARS | N/A | vH+ Con- |
|  |  |  |  | F1SN73 | FYB | N/A | vH+ Con- |
|  |  |  |  | Q9TV77 |  | N/A | vH+ Con- |
|  |  |  |  | I3LVP6 | ANKLE2 | N/A | vH+ Con- |
|  |  |  |  | F1RLS1 | SPECC1L | N/A | vH+ Con- |
|  |  |  |  | M3VH47 | CSNK1D | N/A | vH+ Con- |
|  |  |  |  | I3LFV0 | LOC100628239 | N/A | vH+ Con- |
|  |  |  |  | D3K5M2 | MIOS | N/A | vH+ Con- |
|  |  |  |  | I3LM39 | MB21D1 | N/A | vH+ Con- |
|  |  |  |  | I3LTJ6 | TRRAP | N/A | vH+ Con- |
|  |  |  |  | I3L7M6 | USP33 | N/A | vH+ Con- |
|  |  |  |  | F1RI49 | AP5Z1 | N/A | vH+ Con- |
|  |  |  |  | F1S288 | SNTB1 | N/A | vH+ Con- |
|  |  |  |  | K7GMW4 | SH3KBP1 | N/A | vH+ Con- |
|  |  |  |  | F1SIA3 | ATG13 | N/A | vH+ Con- |
|  |  |  |  | F1RM55 | MAN2A1 | N/A | vH+ Con- |
|  |  |  |  | F1S8T6 | MGEA5 | N/A | vH+ Con- |
|  |  |  |  | F1S2X8 | ANGEL2 | N/A | vH+ Con- |
|  |  |  |  | F1RQH3 | FMNL2 | N/A | vH+ Con- |
|  |  |  |  | F1RL26 | ZC3H7A | N/A | vH+ Con- |
|  |  |  |  | I3LLC0 | SMG8 | N/A | vH+ Con- |
|  |  |  |  | M3TYV9 | TCF20 | N/A | vH+ Con- |
|  |  |  |  | F1S7X0 |  | N/A | vH+ Con- |
|  |  |  |  | I3LLU3 | ZNF132 | N/A | vH+ Con- |
|  |  |  |  | A5H025 | RNASEL | N/A | vH+ Con- |
|  |  |  |  | F1RM23 | CCDC61 | N/A | vH+ Con- |
|  |  |  |  | F1S9Y6 |  | N/A | vH- Con+ |
|  |  |  |  | F2Z587 | HIST1H2AB HIST1H2AE | N/A | vH- Con+ |
|  |  |  |  | I3LUE7 | C5AR1 | N/A | vH- Con+ |
|  |  |  |  | F1RTP0 |  | N/A | vH- Con+ |
|  |  |  |  | I3L5D6 | PLD3 | N/A | vH- Con+ |
|  |  |  |  | F1S3E3 | NHP2 | N/A | vH- Con+ |
|  |  |  |  | Q9TU45 | TYROBP DAP12 | N/A | vH- Con+ |
|  |  |  |  | Q56P28 | ARL6IP5 PRAF3 | N/A | vH- Con+ |
|  |  |  |  | F2Z5V4 | PAFAH1B2 | N/A | vH- Con+ |
|  |  |  |  | F1SKZ8 |  | N/A | vH- Con+ |
|  |  |  |  | F1RIU9 | CHCHD2 | N/A | vH- Con+ |
|  |  |  |  | D2JUK1 | MITF | N/A | vH- Con+ |
|  |  |  |  | I3LMS6 |  | N/A | vH- Con+ |
|  |  |  |  | B8XSK0 | CPNE1 | N/A | vH- Con+ |
|  |  |  |  | Q29076 | POU2F1 OTF1 | N/A | vH- Con+ |
|  |  |  |  | F1SR10 | CMAS | N/A | vH- Con+ |
|  |  |  |  | F1RM12 | LOC100511626 | N/A | vH- Con+ |
|  |  |  |  | F1RSQ6 |  | N/A | vH- Con+ |
|  |  |  |  | F1SD01 | CSTF2T | N/A | vH- Con+ |
|  |  |  |  | F1RUL1 | RBM17 | N/A | vH- Con+ |
|  |  |  |  | Q9GLE3 | CTSK | N/A | vH- Con+ |
|  |  |  |  | F1RL77 | CSF1R | N/A | vH- Con+ |
|  |  |  |  | F1S6R5 | WDR18 | N/A | vH- Con+ |
|  |  |  |  | F1SJQ4 | POLDIP3 | N/A | vH- Con+ |
|  |  |  |  | K9IVK9 | ELF1 | N/A | vH- Con+ |
|  |  |  |  | F1SV59 | ADPRHL2 | N/A | vH- Con+ |
|  |  |  |  | F1RYT3 | SCARB2 | N/A | vH- Con+ |
|  |  |  |  | F1S4D5 | CCBL2 | N/A | vH- Con+ |
|  |  |  |  | F1SGC8 | ACTL6A | N/A | vH- Con+ |
|  |  |  |  | I3LRV0 | LOC100739114 | N/A | vH- Con+ |
|  |  |  |  | F1S912 | FKBP8 | N/A | vH- Con+ |
|  |  |  |  | F1S3K5 | RAVER1 | N/A | vH- Con+ |
|  |  |  |  | F1SHE8 | WDR12 | N/A | vH- Con+ |
|  |  |  |  | F1RRK2 | LOC100517325 | N/A | vH- Con+ |
|  |  |  |  | Q69DL0 | C1QA | N/A | vH- Con+ |
|  |  |  |  | I6NCL0 | NAP1L4 | N/A | vH- Con+ |
|  |  |  |  | F1SH84 |  | N/A | vH- Con+ |
|  |  |  |  | F1RVY0 | TOR4A | N/A | vH- Con+ |
|  |  |  |  | F1RTZ5 | LOC100524801 | N/A | vH- Con+ |
|  |  |  |  | F1SBT9 | MLLT1 | N/A | vH- Con+ |
|  |  |  |  | I3LAN0 | ZEB2 | N/A | vH- Con+ |
|  |  |  |  | A5GFU0 | GNAS CH242-247L10.1-017 | N/A | vH- Con+ |
|  |  |  |  | F1S2Z5 |  | N/A | vH- Con+ |
|  |  |  |  | F1SLI6 |  | N/A | vH- Con+ |
|  |  |  |  | F1SU88 |  | N/A | vH- Con+ |
|  |  |  |  | F1RQY4 | UBTF | N/A | vH- Con+ |
|  |  |  |  | I3L9C9 | SON | N/A | vH- Con+ |
|  |  |  |  | I3LES5 | DUS2 | N/A | vH- Con+ |
|  |  |  |  | F1S6P9 | GATAD2A | N/A | vH- Con+ |
|  |  |  |  | I3L7B7 | DDX18 | N/A | vH- Con+ |
|  |  |  |  | F1SAB0 |  | N/A | vH- Con+ |
|  |  |  |  | E7EI18 |  | N/A | vH- Con+ |
|  |  |  |  | I3LV06 | DDX59 | N/A | vH- Con+ |
|  |  |  |  | F1RKB5 | LOC100512219 | N/A | vH- Con+ |
|  |  |  |  | F1SPI2 | XPC | N/A | vH- Con+ |
|  |  |  |  | F1RGE2 | IK | N/A | vH- Con+ |
|  |  |  |  | F1RS45 | TOP2B | N/A | vH- Con+ |
|  |  |  |  | F1SFG0 | NIN | N/A | vH- Con+ |
|  |  |  |  | F1RY51 | KIAA1429 | N/A | vH- Con+ |
|  |  |  |  | I3LMC6 | LOC100523938 | N/A | vH- Con+ |
